# Recent land use and management changes decouple the adaptation of livestock diversity to the environment

**DOI:** 10.1038/s41598-020-77878-2

**Published:** 2020-12-03

**Authors:** Elena Velado-Alonso, Ignacio Morales-Castilla, Antonio Gómez-Sal

**Affiliations:** 1 FORECO – Forest Ecology and Restoration Group, Department of Life Sciences, Universidad de Alcalá, Ctra. Madrid-Barcelona Km 33.600, 28805 Alcalá de Henares, Madrid, Spain; 2 GloCEE - Global Change Ecology and Evolution Group, Department of Life Sciences, Universidad de Alcalá, Ctra. Madrid-Barcelona Km 33.600, 28805 Alcalá de Henares, Madrid, Spain; 3 Department of Environmental Science and Policy, George Mason University, 4400 University DriveDavid King Hall Rm 3005, Fairfax, VA 22030-4444 USA

**Keywords:** Agroecology, Biodiversity, Ecology

## Abstract

Native livestock breeds, i.e. those autochthonous to a specific region, are locally adapted domesticated animals that conserve genetic resources, guaranty food security and provide agroecosystem services. Native breeds are largely threatened worldwide by agricultural intensification and rural areas abandonment processes related to recent changes in production schemes and planning. Yet, our gap of knowledge regarding livestock breed-environment relationships may prevent the design of successful conservation measures. In this work, we analyse the links between livestock diversity -i.e. richness of native breeds- and a selection of environmental factors that express at broad scales, with a temporal perspective. We compare native breeds distributional patterns before and after the agricultural intensification, in the context of land-use change in mainland Spain. Our results confirm the existence of strong associations between the distribution of native livestock breeds and environmental factors. These links, however, weaken for contemporary distributions. In fact, changes in breed distribution reflect a shift towards more productive environments. Finally, we found that the areas having higher breed richness are undergoing land abandonment processes. Succeeding in the conservation of threatened native breeds will require going beyond merely genetic and production-oriented views. Ecological and sociocultural perspectives should also be accounted for as global change processes are determinant for livestock agrobiodiversity.

## Introduction

Understanding the distribution of biodiversity is a major goal of ecology. An extensive literature has proposed numerous hypothesis to explain biodiversity gradients, usually linked to environmental factors such as ambient energy, water availability, vegetation productivity or environmental heterogeneity^1,2^. Most of this work has focused on wild species, while much less is known about the distribution of agrobiodiversity -i.e. the variation within and across agricultural plants and domesticated animals-, especially in the case of livestock^3,4^. Yet, knowing which factors underlie the distribution of agrobiodiversity would be critical to understand the adaptation processes responsible to generate it and to plan conservation actions where needed.

Native livestock breeds are those autochthonous and locally adapted to a specific region^5^. They are intraspecific groups with identifiable inheritable external traits^6^, resulted from differentiation processes of domesticated animals^7,8^. They are regarded to as geographically and/or culturally distinct and they are supported and maintained by a community of breeders. In these processes, human intended and non-intended selection, as well as other factors such as genetic and geographic isolation, inbreeding and genetic drift, ecological and historical processes or human geography, have been the key to create and maintain breeds over time^5,9,10^. Thus, both natural and artificial selection are involved in the diversification of breeds^9^. In addition, livestock breeds are considered as management and conservation units of livestock agrobiodiversity^11,12^.

For all that, native livestock breeds represent important ecocultural -i.e. culturally and environmentally mediated- entities to preserve^13^. First, they help maintaining the diversity of animal genetic resources and thus guaranty food security^13,14^. Second, their conservation prevent the loss of rare and unique phenotypes of current or potential future importance^15^. Third, breeds act as driver and providers of agroecosystem services^16^, which are expected to be secured or increased with higher diversity rates. However, livestock breed diversity is largely threatened. Currently, there are 7,136 livestock breeds that occurs only in one country catalogued by FAO worldwide, of which 27% are endangered and 65% have an unknown status^17^.

Even if the value of locally adapted livestock breeds is largely recognised^18^, major shortfalls in our knowledge about them remain. For example, although breeds being highly locally adapted is claimed as a chief reason for their worth, especially in the context of climate change^19^, there are still gaps in our knowledge about the mechanisms involved in breeds adaptations^3,20^. However some efforts have been done in that direction in recent decades^21–23^. Besides, even when in situ is supposedly the preferred conservation option^24^, most research focuses on breed genetics and animal production. For the moment, research on the topic from an ecological perspective has been neglected^19^, and even so, ecological views could help to widen our understanding of breed-environment interactions.

Wild and domesticated diversity have followed markedly different evolutionary pathways^25^. While climate and biogeography have proven to exert a major influence on wild diversity^26^, less is known on their influence on the distribution of domesticated diversity. For example, we know that the distribution of domesticated animals is associated with human migrations, through a complex process where local adaptation and blending with wild populations seems to have been frequent^27^. Wildlife diversity is also known to be limited by human pressures^28,29^, especially in regions deeply modulated by humans such as the Mediterranean basin^30,31^ over a historical process of at least 7,000 years. And yet, there is evidence that bioclimatic factors have determined regions where the genome of livestock breeds would have endured stronger selective pressures^32^.

Environmental heterogeneity has been proposed as a driver of wild species richness, since it would increase the available niche options, enhancing species coexistence, providing refuges, promoting species persistence and increasing the probability of speciation events resulting from isolation or adaptation^2^. In the case of domesticated animals, heterogeneity could have played a similar role, though acting through different ways. Firstly, human needs and human-modified environments should have fostered heterogeneity, promoting diversity among domestication pathways^33^, through processes such as human-animal cultural coevolution^34^. Secondly, during post-domestication specialization processes, prompting the diversification of local and regional populations, responding to new demands in the context of traditional agriculture^8^.

Climatic conditions should have played an additional role in shaping the distributional ranges of domesticated animals based on their physiological requirements and the availability of resources^35^, as in the case of wild species^26^. They should also have had, both direct and indirect effects, on the adaptation of breeds to local environments through physiological mechanisms^36,37^. In addition, the relaxation of intra- and interspecific competition, due to human control of natural selection (e.g. predator pressure), must have facilitated the emergence of new phenotypes^38^.

Conversely, human factors -e.g. diversity of production systems, agricultural area and land cover types- are positively correlated with the number of breeds reported by each country^39^. Nonetheless, human factors have been also identified as core drivers of livestock diversity erosion, mainly related to recent agricultural intensification, due to abandonment or replacement with highly productive breeds, crossbreeding or lack of economic profitability of the native breeds^3^. The conservation of livestock diversity is largely threatened by under-utilization, contrary to the case of wild diversity which usually is more related to overuse, e.g. overhunting or habitat degradation ^19^. In this context, the processes of land-use intensification and abandonment, which usually occur in parallel as a manifestation of global change^40^ are key factors that currently threaten (or at times foster) wildlife diversity^41^. These processes might also be affecting the management and contemporary distribution of livestock breed diversity^3^, since land use changes are mainly related to agricultural practices.

The goal of this work is twofold. First, to quantify changes in the associations between the distribution of livestock agrobiodiversity -i.e. different estimates of local breed richness, referring as local breeds those autochthonous locally adapted—and environmental factors in mainland Spain, a remarkable area of livestock breeds richness in Europe. We hypothesise that at broad scales, livestock breed richness respond to environmental factors (mostly climate) according to each species physiological requirements, analogous to what is observe for wild vertebrates biodiversity^42^. This is, based on the water-energy hypothesis^26^, we expect positive relationships between local livestock breed richness and predictors such as temperature and precipitation^26^. Second, we explore how the current distribution of livestock diversity relates with land-use changes occurred in the last decades. We determine where in the current land use context is more likely to find higher richness of local livestock breeds. We hypothesise local Spanish breed present distribution relates to rural abandonment, and is affected by agricultural intensification^3^.

### Results

Our results show a major effect of environmental factors on the distribution of local livestock breed diversity both for past –i.e. before agricultural intensification- and current distributions –i.e. after agricultural intensification (see methods for more detail). This pattern is robust across all the studied domesticated species. Environmental factors explain up to three quarters of the variation in species diversity for past distributions (i.e. from global quasi-R^2^_ovine_ = 0.39 to global quasi-R^2^_equine_ = 0.73; see Table 1), and up to half the variation for current distributions (i.e. from global quasi-R^2^_ovine_ = 0.20 to global quasi-R^2^_bovine_ = 0.43; see Table 1). Consistently, environmental factors explain more variation in total breed richness for the past distributions than for contemporary distributions of livestock (global quasi-R^2^_past_ = 0.64; global quasi-R^2^_present_ = 0.46; see Table 1). This pattern of stronger associations between the environment and past distributions is constant across species –i.e. bovine, ovine, caprine, equids, porcine (see Table 1)-, sampling grain size -i.e. 10 × 10, 20 × 20 and 50 × 50 km (see Table 2, more detail in SI, Appendix 2-I)- and analysis extent -i.e. 2.5%, 5%, 10%, 20% of data bandwidth (see methods section). These results are also robust to a sensitivity test excluding extinct or new local breeds from the analysis (for more details see SI, Appendix 2-III).

**Table 1:** Global quasi R^2^ values of the Geographically Weighted Regression fitted models, with an analysis extent of 5% of the total data as bandwidth, for each studied livestock species richness (i.e. bovine, ovine, caprine, equid -horses and donkeys-, porcine and total, sampled at 10 × 10 km UTM grid cell), in past and present distributions, using as predictors: annual mean temperature, annual precipitation, precipitation seasonality and vegetation productivity seasonality (see more detail in SM Appendix 2).

	Global Quasi-R^2^	
	Past	Present
Bovine	0.63	0.45
Ovine	0.39	0.20
Caprine	0.40	0.29
Equid	0.73	0.41
Porcine	0.66	0.35
Total	0.64	0.46

**Table 2:** Global quasi R^2^ values of the Geographically Weighted Regression fitted models with an analysis extent of 5% of the total data as bandwidth, considering different sampling scales (20 × 20, 50 × 50 km UTM grid cell) for each studied livestock species richness (i.e. bovine, ovine, caprine, equid -horses and donkeys-, porcine and total) in past and present distribution, using as predictors annual mean temperature, annual precipitation, precipitation seasonality and vegetation productivity seasonality (see more detail in SM, Appendix 2).

	Sample size	Global Quasi-R^2^	
		Past	Past
Bovine	*20* × *20*	0.65	0.37
	*50* × *50*	0.66	0.48
Ovine	*20* × *20*	0.43	0.16
	*50* × *50*	0.57	0.24
Caprine	*20* × *20*	0.43	0.23
	*50* × *50*	0.46	0.35
Equid	*20* × *20*	*0.73*	*0.42*
	*50* × *50*	*0.75*	*0.47*
Porcine	*20* × *20*	0.66	0.24
	*50* × *50*	0.70	0.38
Total	*20* × *20*	0.65	0.41
	*50* × *50*	0.68	0.51

**Figure 1:**
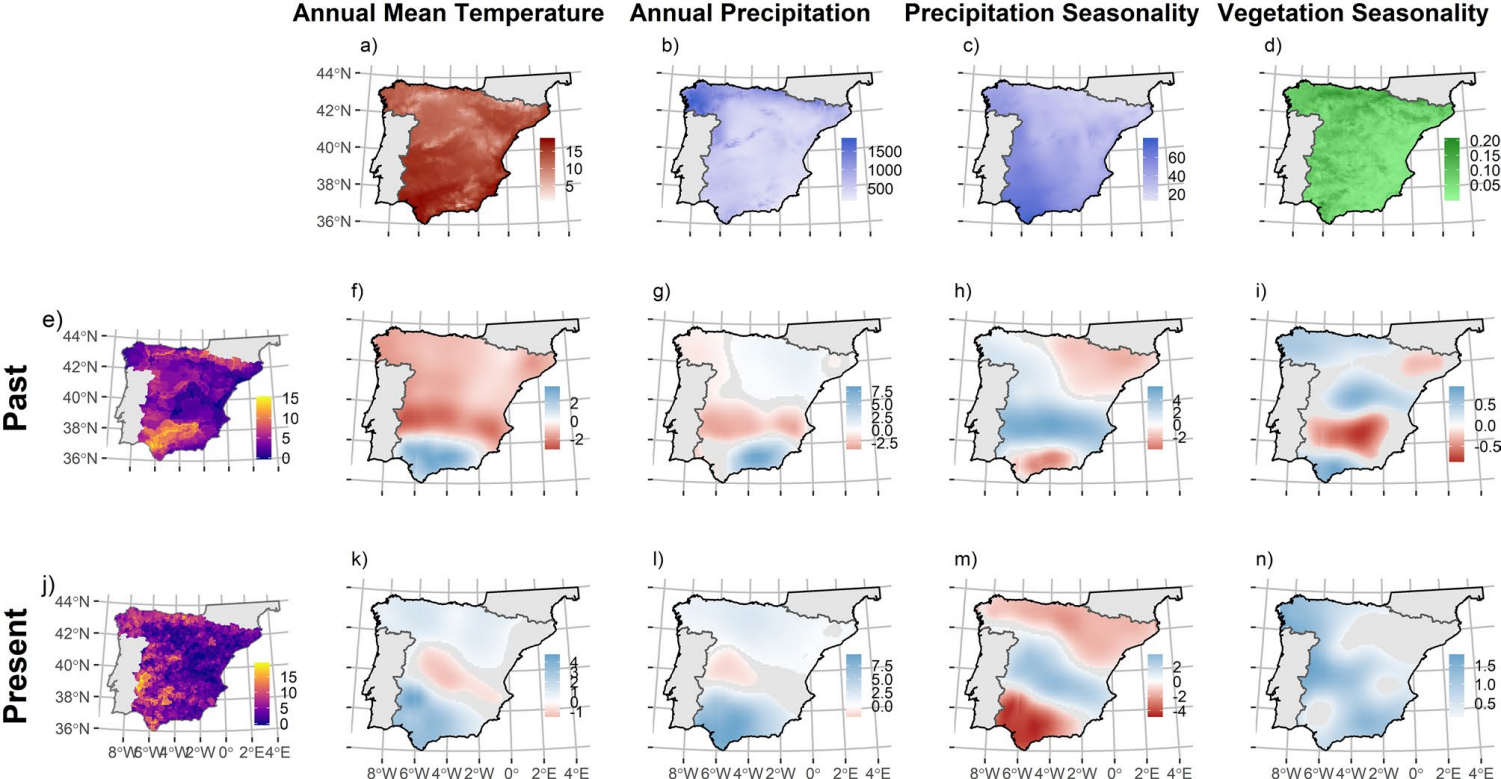
Maps of regression coefficients (surface of predictions) resulting from Geographically Weighted Regression models from past (**f**–**i**) and present (**k**–**n**) periods of time, fitting the relationships between total native breed richness, i.e. number of breeds per cell-sampled at 10 × 10 km UTM grid cell-, for past (**e**) and present (**n**) distributions, using as predictors annual mean temperature (**a**), annual precipitation (**b**), precipitation seasonality (**c**) and vegetation productivity seasonality (**d**). Depicted coefficients are only coloured when statistically significant at [*P* = 0] ≤ 0.05. Blue colour represents positive coefficients and red colour represents negative associations. Figure was created using “sf” and “ggplot2” packages in R v3.6.0 software (https://www.R-project.org/).

The distribution of local livestock breed diversity has changed over time and so has its associations with environmental factors (see Fig. 1). Overall, when considering total breed richness, the distribution has shifted from hotspot areas southern part of the studied area (Guadalquivir basin and surrounding mountains), and the north-eastern part of mainland Spain (Pyrenees Mountain range), to areas placed in south-western and western Spain, close to Portuguese border, and the Atlantic regions, north and north-western Spain (see Fig. 1e, j).

GWR models show a contrasting effect of environmental factors on the past and present distribution of total local breed richness. In general, past breed richness is negatively, neutral or non-significantly associated to temperature and precipitation across most of the studied territory (Fig. 1f, g). There is an exception in the southern corner of Spain, where positive coefficients coincide with the diversity hotspot of Guadalquivir basin (Fig. 1e). In contrast, the contemporary distribution of livestock diversity is positively associated with these environmental factors across most of the Spanish geography (Fig. 1k, l). The climatic seasonality seems to have had general positive effects on livestock richness distribution in the past (Fig. 1h, i). Precipitation seasonality presents higher positive coefficients in central mainland Spain where intermediate local breed richness values are found (Fig. 1e), and the seasonality of vegetation productivity shows greater positive coefficients in the central-north and central eastern Spain, coinciding with the Iberian mountain range and central plateaus, where, due to altitude and climate continentality, vegetation productivity is quite unpredictable^43^. In the contemporary distributions, the association with the seasonality of vegetation productivity remains positive (Fig. 1n), but precipitation seasonality becomes strongly negatively associated over northernmost and southernmost ends of mainland Spain (Fig. 1m). Coarse extent analyses -i.e. 20% of data as bandwidth- show that the four environmental factors become positively associated to contemporary total livestock breed richness (for more details see SI, Appendix 2-II, Fig. S1).

**Figure 2:**
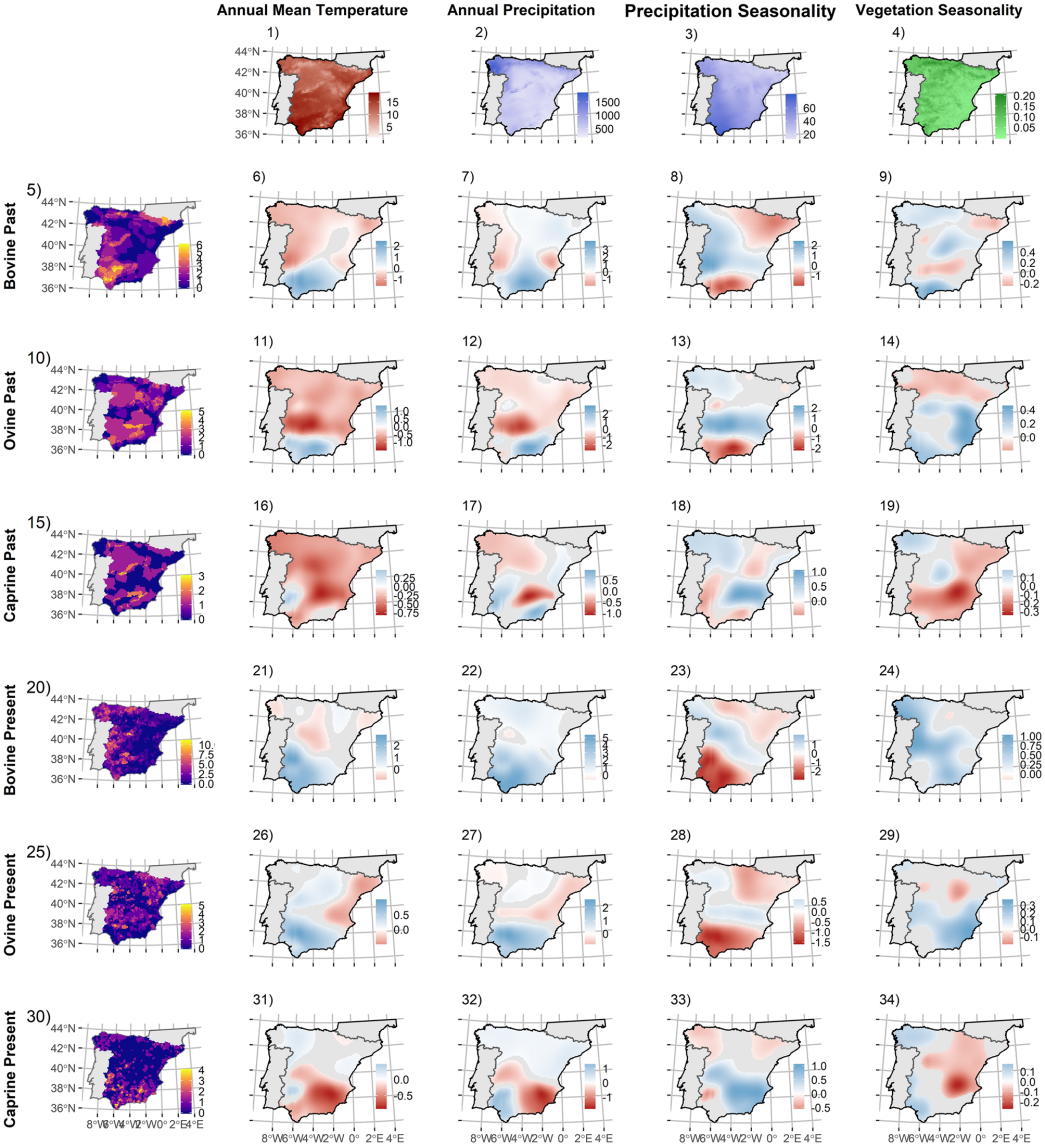
Maps of regression coefficients (surface of predictions) resulting from Geographically Weighted Regression models using 5% of the data as bandwidth, fitting the relationships between equid- horses and donkeys-(5, 10) and porcine (15, 20) native breed richness (sampled at 10 × 10 km UTM grid cell) for past (5–9, 15–19) and present distributions (10–14, 20–24), using as predictors annual mean temperature (1), annual precipitation (2), precipitation seasonality (3) and vegetation productivity seasonality (4). Depicted coefficients are only coloured when statistically significant at [*P* = 0] ≤ 0.05. Blue colour represents positive coefficients and red colour represents negative associations. Figure was created using “sf” and “ggplot2” packages in R v3.6.0 software (https://www.R-project.org/).

**Figure 3:**
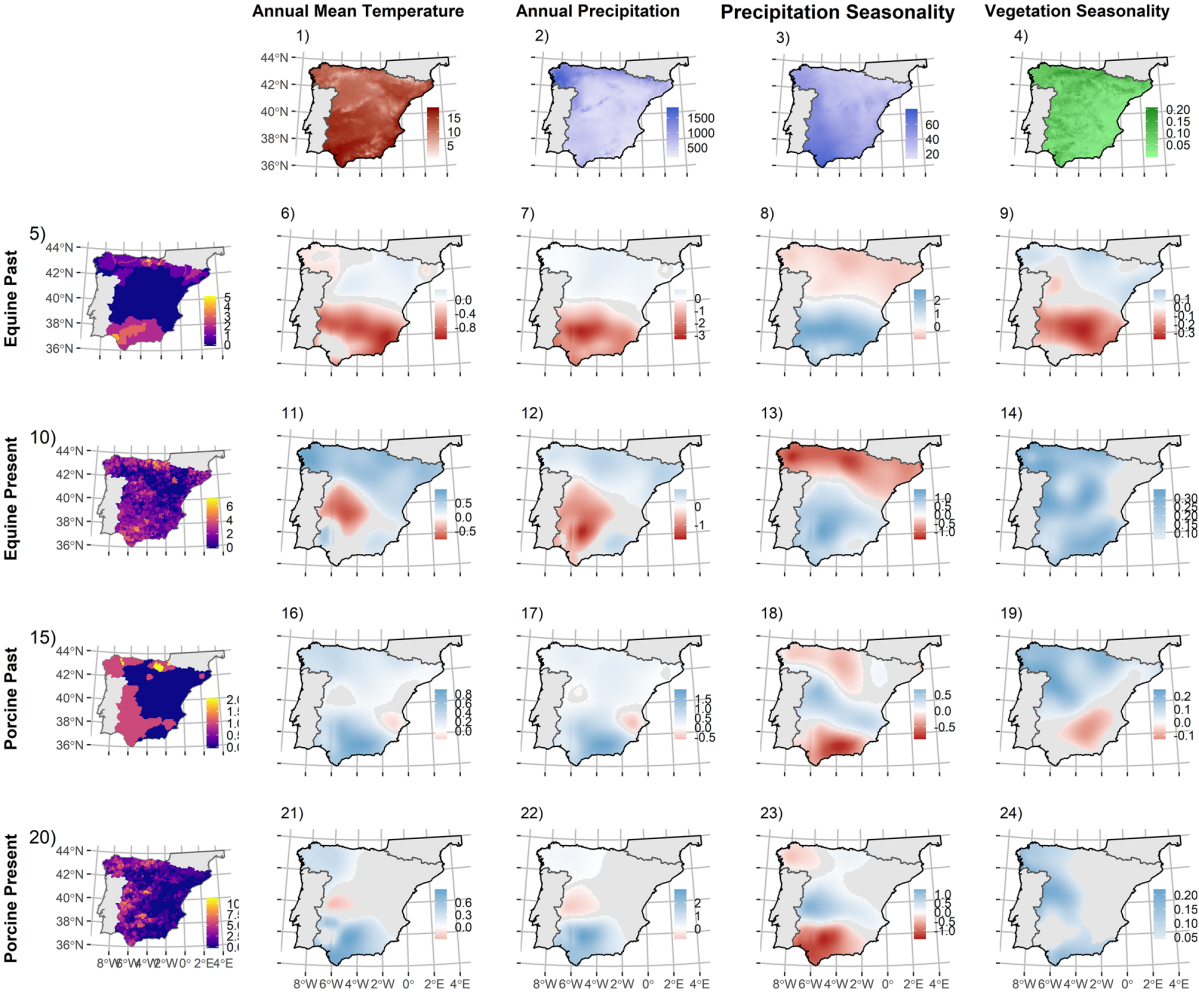
Maps of regression coefficients (surface of predictions) resulting from Geographically Weighted Regression models using 5% of the data as bandwidth, fitting the relationships between bovine (5, 10), ovine (10, 25) and caprine (15, 30) native breed richness -sampled at 10 × 10 km UTM grid cell- for past (5–19) and present (20–34) distributions, using as predictors annual mean temperature (1), annual precipitation (2), precipitation seasonality (3) and vegetation productivity seasonality (4). Depicted coefficients are only coloured when statistically significant at [*P* = 0] ≤ 0.05. Blue colour represents positive coefficients and red colour represents negative associations. Figure was created using “sf” and “ggplot2” packages in R v3.6.0 software (https://www.R-project.org/).

GWR models fitted separately for breed diversity of each studied species reveals contrasting patterns. In general, ruminant species -i.e. bovine, ovine and caprine- present a common pattern where mean temperature, annual precipitation and seasonality of the vegetation productivity are mostly negative or invariant in their associations with the past distribution of breed richness (Fig. 2^5–19^). Regarding contemporary distributions, stronger positive coefficients are found for the environmental factors, except for precipitation seasonality, in bovine and ovine, but not in caprine species distribution (Fig. 2^20–34^). On the contrary, the associations of the distributions of porcine and equine breed richness with environmental factors remain invariant regardless whether past or present distributions are considered. Mean temperature, annual precipitation and seasonality of the vegetation productivity are mostly positive for porcine breeds, whereas for equine breeds are positive northern Spain and negative southern Spain (Fig. 3). These patterns change for precipitation seasonality, which shows positive associations southwards and negative northwards (Fig. 3). Similarly, to the patterns for all livestock species together, as analyses use coarser extents, differences across species weaken and tend to become positive for contemporary distributions (see SI, Appendix 2-II, Fig. S2–S6).

**Figure 4:**
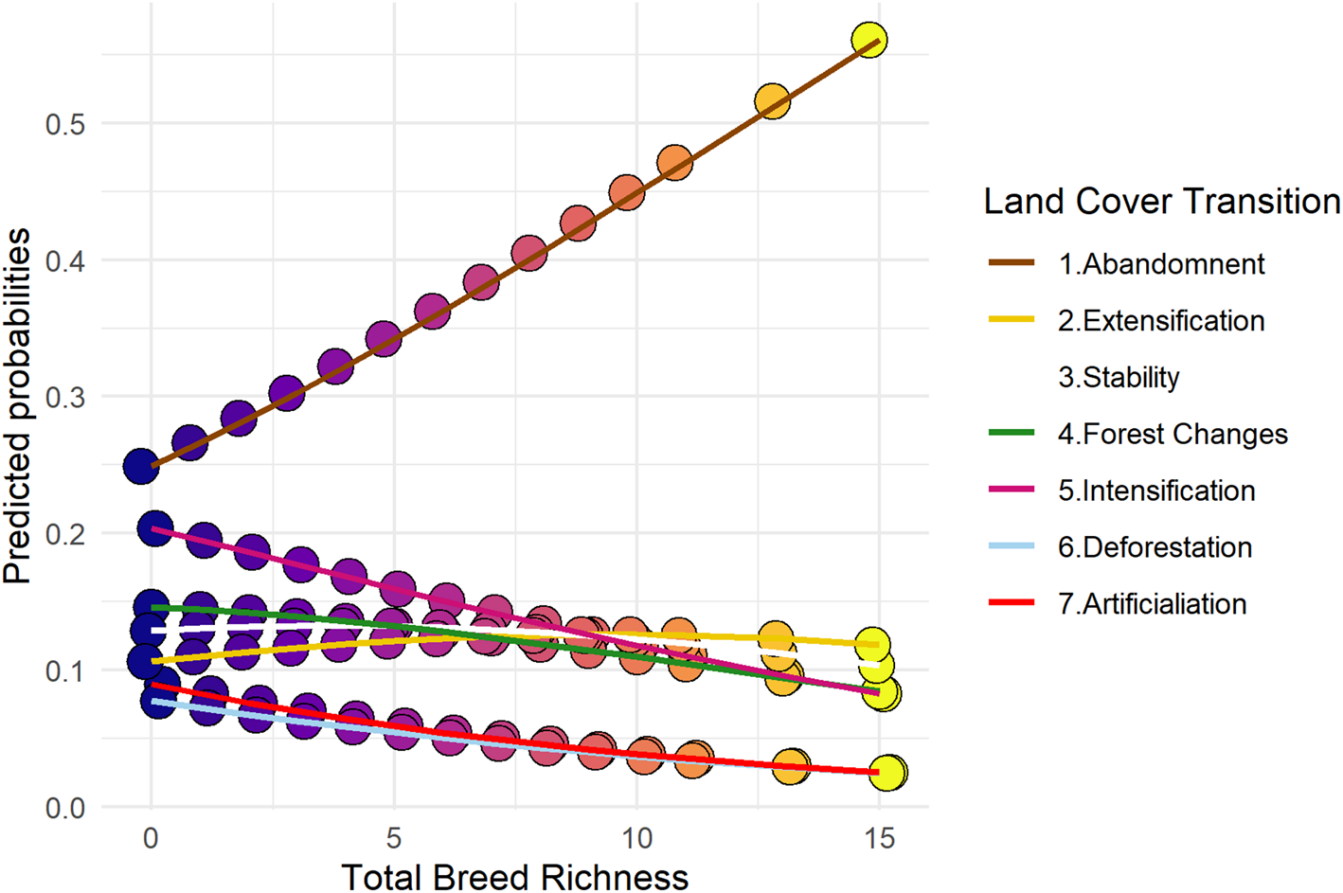
Predicted probabilities of total native breed richness -based on current distribution by municipality- (from 0 to 15) in each land cover transition class (see legend), calculated from Ordinal Logistic Regression model (consult SI Appendix 3 for more detail).

The results about current local breed distribution related to land use changes show that livestock breed richness is higher in areas undergoing agricultural farm abandonment. Our models -e.g. Ordinal Logistic Regressions, OLR- show that municipalities experiencing afforestation linked to farm abandonment -i.e. land-use change class 1- are twice more likely to harbour high than low diversity of breeds (Fig. 4). When compared against municipalities undergoing different land-use processes -i.e. land-use change classes 2 to 7, ranging from farm extensification to artificialization-, the former is up to three times more likely to have higher livestock breed richness (see Fig. 4). In locations going through agriculture extensification -i.e. class 2- it is slightly more likely to find high local livestock breed richness. In contrast, within municipalities subjected to agricultural intensification -i.e. classes 3 to 7- finding high breed richness is decreasingly probable (Fig. 4). These patterns are robust when considering the studied species separately, except for equids, where ORL models are not significant (SI, Appendix 3).

## Discussion

Our results show that, despite the major role played by humans on the differentiation and distribution of livestock breeds^9^, the environment also has an imprint on the distribution of livestock diversity. The distribution of livestock agrobiodiversity is significantly associated to environmental factors, but the strength of the associations has decreased for contemporary distributions. As proven for wildlife diversity and hypothesized for agrobiodiversity, variables linked to water-energy hypothesis seem to be suitable predictors to explain both distributions. However, we find that the direction of model coefficients has shifted with time. Overall, the areas where past distribution -i.e. before agricultural intensification- was negatively associated with mean temperature and annual precipitation, become positively or not associated in the present (more so at coarser scales, see SI, Appendix 2-II). This pattern indicates that (a) the past distribution of livestock diversity was associated to low productive regions, usually with higher climatic seasonality and topographic heterogeneity, (b) the current distribution tends to occupy more productive environments i.e. areas with higher mean temperature and annual precipitation. In addition, the highest values of native breed richness are found in areas undergoing farm abandonment processes. Taken together, our results confirm that beyond historical or present human determinants -e.g. migrations, artificial selection, type of production system or economically oriented management criteria- the distribution of livestock breeds was (and remains) highly subjected to environmental conditions.

The relationships between environmental factors and wildlife diversity has been amply studied^1,2,26,44,45^, but they have rarely been explored for livestock diversity^46,47^. However, new insights from research in landscape genetics and selection signatures point out to biogeographical factors such as climate^32,36,37^, as well. Our study pioneers the attempts to quantify the effect of environmental factors on the distribution of livestock agrobiodiversity from a biogeographic perspective and including several domesticated species. However, our approach faces limitations. On one hand, the lack of higher resolution in breeds distributional data so it can be tied to more precise periods of time or to the demography of livestock breeds. On the other hand, the breed concept has a fundamental human dimension and is strongly connected with farm environment and anthropogenic selection^47,48^. That leads for example to changes in breed recognition and management through time, which in the case of Spain has prompted the official recognition of new breeds that were formerly considered varieties. We have circumvented these limitations by considering time dynamics -i.e. analysing breed distributions before and after agricultural intensification-, and by conducting exhaustive sensitivity analyses -i.e. at multiple grain sizes or resolutions, multiple analysis extents, and varying breed grouping approaches. The observed patterns are robust regardless the approaches (see SI, Appendix 2).

Predictors linked to water-energy hypotheses explain much of the distribution of local livestock breed richness (see Tables 1–2) but the underlying mechanisms may differ from those determining wild diversity distributions. For example, diversity gradients for wild species commonly co-vary with temperature and water availability positively -i.e. increasing productivity^26,49^ (but see^50^)-, but past distributions of livestock diversity were negatively associated with these predictors in large portions of the Northern half of Spain (see Figs. 1,2,3,4; see SI, Appendix 2 for more details) and in general positively associated with vegetation productivity seasonality. This is likely due to domesticated livestock species were forced to adapt to suboptimal or less favourable environment as humans broaden their distribution beyond their original environmental limits, linked with the expansion outside their domestication areas^27,35^.

It stems that past distributions of livestock diversity were kept away from their supposed *climatic equilibrium*^51^ -i.e. possibly a reason underlying intra-specific diversification, as adaptation to new and challenging environments^5,52^-, while the weaker associations between current distributions and climate (Table 1) would suggest that livestock diversity is undergoing a redistribution process strongly conditioned by human options, in the context of current land-use change. The modernization of farming systems is determining to what extend livestock animals are exposed to natural conditions and fed from local resources, especially under intensive management, but also in many livestock systems considered as extensive^53,54^.

Observed shifts on the tendency of environmental factors at present distribution suggest that this distribution occupies more productive environments than before. A small number of breeds currently spreading and increasing in number are playing a main role in the geographic reshuffle of livestock diversity posterior to agricultural intensification. For example, breeds from bovine and porcine species, such as “*Rubia Gallega*” or “*Asturiana*” cattle, or the recognition as breed of former varieties of “*Iberico*” pig, are nowadays associated with more intensive farming systems —for example with the abandonment of traditional grazing locations, the increasing of herd size or with the supply of concentrates^55–57^— and high-quality products, such as gastronomic specialities like Iberian ham. At the same, the area occupied by “*Pura Raza Español*” horse has spread linked with professional sport and leisure activities. Thus, their breeding is nowadays highly specialised and more independent of natural conditions and local vegetation resources.

The livestock sector has suffered a rapid transformation during recent decades. The increasing demand of livestock products has triggered the “livestock revolution” and the expansion of more homogenous and industrialised livestock systems^58^. A process that has also affected breed distributions in Spain (see Fig. 1 and SI, Appendix 2-II). Thus, this observed changes on breed distribution can be interpreted as a result of increasingly anthropic pressures, relaxing breed-environment interaction and changing the nature of this relationship. This fact represents a threat to domesticated animal diversity conservation. On the one hand, intensification of faming systems and separation of the traditional environments of breeds could diminish they adaptive ability to local and challenging environments, which is one of the reason given to their acknowledged value^13^. On the other hand, the intensification of farming systems and the separation of livestock breeds from the environment could break the adaptation processes to land-based production systems, triggering evolvability of native breeds to tightly controlled artificial environments, or at least to fewer challenging environments.

Finally, two major opposite processes of land-use change -i.e. farm abandonment and intensification of the agricultural production systems- are related to the distribution of livestock agrobiodiversity and thus, should be accounted for by any conservation efforts. Areas of abandonment coincide with livestock diversity hotspots (Fig. 4). In contrast, areas of intensification, known to compromise the conservation of natural habitats and wild species^59–61^, also have a negative impact on livestock agrobiodiversity^19^ (Fig. 4).

Spanish native livestock breeds were associated with traditional farming systems, bred with different types of natural or agricultural vegetation (pasturelands, silvopastoral systems, stubble, etc.). Moreover, they have played a crucial role in the economy of most Spanish rural areas^43,62^. The abandonment of these land-based livestock systems is transforming the landscape. The fact that more than 80% of the studied Spanish breeds are currently at risk according to criteria by the Spanish Ministry of Agriculture^63^, indicates a reduction of extensive and traditional livestock activities, favouring the transformation of agricultural landscapes into natural areas. Unfortunately, such reduction is not necessarily joint by the enhancement of suitable strategies of wilderness and nature conservation. Within these abandonment areas, native breeds concentrate in sites with greater primary productivity. Our finding suggests a break between local breeds and their associate environments, as even in areas with high breed richness the abandonment processes tend to dominate. This calls for conservation actions to avoid breed extinction, associated with sustainable development based on local resources and the conservation of wildlife^64^.

Our results highlight the importance of both environmental and human factors on the distribution of native livestock breed. This study moves forward previous works to document livestock agrobiodiversity-environment interaction from an ecological perspective, however further efforts are needed. On the one hand, it is important to understand how global change would affect livestock agrobiodiversity since two major components -i.e. climate change and land use change- could be determinant on native livestock breeds. On the other hand, breed conservation planning needs to integrate the processed described in this work, changing to more holistic perspective of livestock conservation and management that expand the focus from genetic and productive to also ecological and sociocultural dimensions.

## Methods

### Distributional and environmental data: local livestock breeds in mainland Spain

The area of study is mainland Spain, located in the Iberian Peninsula. This is a territory characterised by old agricultural uses —for at least the last millennium^30^— and great heterogeneity of landscapes^43^, that have led to a significant agrobiodiversity. Livestock activities in mainland Spain have had historical -there are archaeological evidences of livestock activities since 7,500 years^27^-and economic importance^62^, driving the differentiation of a substantial number of local livestock breeds^63^, i.e. autochthonous or native. For the present work we have used all extant (118) and extinct (15) local breeds from the bovine, ovine, caprine, asinine, equine and porcine species in mainland Spain. We used the breed classification list from the Spanish Official Catalogue of Livestock Breeds^63^ to include all currently identified and recognised local breeds. All these breeds are supported by an Official Breeding Association, which is responsible for the management of the Herding Book. In addition, we selected those breeds listed as extinct in the FAO Domesticated Animal Diversity-Information System^65^ that were also mentioned in the Spanish livestock breed literature and recognised as Spanish autochthonous breeds —but currently lack of a Breeders Association. In total, they represent 133 breeds: 44 bovine, 38 ovine, 19 caprine, 4 asinine, 14 equine and 14 porcine (SI, Appendix I, Table S1).

To determine the geographic distribution of each breed over time we follow two different pathways. First, to stablish the distribution before agricultural intensification, as there are not available data on the dynamics of breed distributions over time, we identify the area of origin of each breed through a literature review of the main catalogues of Spanish breeds (SI, Appendix 1, Table S2). We considered as area of origin the zone where each breed was first described, claimed as origin area. If that was not clear, we assigned the oldest region of distribution, except for the new Ibérico porcine breeds. These were in the past considered as varieties and any specific information was found for them. We assume that the areas of origin represent closely the distribution of local breeds before agricultural intensification, representing the historical and eco-cultural territory of each breed^66^. Areas of origin were digitally mapped using QGIS 2.18.26 “Las Palmas” software^67^.

To determine the current geographic distribution, as it is only available in detailed resolution at administrative NUTS 3 units, we used the information of the National Programme for the Conservation, Improvement and Promotion of the Spanish Livestock Breeds. We collected all the farm identification numbers of those farms that were collaborating with the conservation program during the period 2017–2019. This information was provided by different sources: mainly by the National Breed Information System of the Spanish Agriculture Ministry and some Autonomous Community Administrations. In cases where the administration could not offer data on the breeds we requested; we would directly contact with the Breeding Associations. Lastly, for four specific breeds which was impossible to collect any information by this mean, we used the listed farms available on their specific conservation program, that did not precede the year 2015 of publication. Only one porcine local breed information was impossible to collect, the one for Euskal Txerria pig.

The first five digits of the farm identification number correspond to the municipality (LAU2 administrative level). By this way we mapped the areas of distribution in the present, i.e. after agricultural intensification, of Spanish local livestock breeds by municipalities using R software^68^. For those breeds categorised as at risk by Spanish ministry (more than 80% of studied breeds), 100% of farms are collaborating with the conservation program, however for those increasing in number (less than 20%) that is not the case. To see the percentage of farms included in the study for those increasing in number local breeds see SI, Appendix 1, Table S3.

Then, for both present and past distributions, we calculated several richness indices (for each species and total livestock breeds), considering richness as the sum of all breeds present in each UTM grid cell. Since present distributions have a finer scale -i.e. municipalities- than past distributions -i. e. based on areas of origin-. We calculated the richness indices at 3 different scales, i.e. 10 × 10, 20 × 20 and 50 × 50 km UTM sampling grain, in order to test data scale bias.

In addition, to stablish to what degree the distribution of local livestock breed is determined by environmental factors, we calculated average values of a suite of variables within the 10 × 10, 20 × 20 and 50 × 50 km UTM cell. We used annual mean temperature, annual precipitation, precipitation seasonality and vegetation productivity seasonality. Climatic data was obtained from the 30 s BIO1, BIO12 and BIO15 layers of WorldClim version 2^69^ and vegetation productivity seasonality was calculated from the coefficient of variation of the Enhanced Vegetation Index (satellite-derived Ecosystem Functional Attributes)^70,71^ on the basis of the Global MOD13Q1 for the 2001–2017 period. That descriptor has been utilised to study diversity richness in mainland Spain at similar scales^72^. We have used the same environmental data for the two broadly defined periods of time. Past distributional data lacks information on breed origination times preventing to link past distribution to specific past environmental data. The underlying assumption is that, at the scale used here, environmental factors would have remained relatively stable across periods. Such assumption may be partially supported by the effects of global warming manifesting strongly only after the 1980s^73–75^.

The descriptors were chosen as they help to characterise the water-energy dynamics on the system, are good indicators of primary productivity in warm and dry climates as the Mediterranean^26^, as well as being dynamic variables that are expected to change under the ongoing global change^73^. All variables were standardised through normalization, in order to improve the interpretability and facilitate the comparison within and between models^76^.

Lastly, in order to explore the relationship between the current distribution of local breeds and land use changes, we used the map proposed by Fernández-Nogueira and Corbelle-Rico of land cover transitions based on Corine Land Cover in 1990, 2000 and 2012 by municipalities in Spain (LAU2 level)^77^. In order to facilitate the interpretation of these results, we have classified the transitions from less to more intensified, based on the dominant transition in the municipalities from 1.Abandonment -those municipalities where afforestation dominates, related to agricultural abandonment in tension with conversion to agriculture-, 2.Extensification -agriculture extensification-, 3.Stability -municipalities where stability along the 22 years period dominates-, 4.Forest Changes -afforestation and changes on forest composition-, 5.Intensification -agricultural intensification-, 6.Deforestation -deforestation-, to 7.Artificialization -increase of urban areas. Finally, we calculated current local breed richness indices in each municipality.

### Statistical analysis

To test to what extent environmental factors are determining the distribution of local livestock breeds richness distribution, we performed a set of Geographically Weighted Regression models (GWR), a frequent technique used to modelling spatial non-stationarity on the distribution of wildlife^78–80^. This GWR fits a regression considering each spatial unit with the geographically weighted (based on a distance function) neighbouring units up to a given bandwidth, i.e. analysis extent. GWR models allow to identify spatial shifts in the direction of the associations among response and predictor variables, taking into consideration the spatial variation (non-stationarity)^81,82^.

Firstly, to test possible effects of sampling scale we performed a set of GWR for each domesticated species and total local breed richness indices, using an adaptive bandwidth including 5% of the spatial units in our dataset (i.e. ca. 100 km bandwidth), for the 10 × 10, 20 × 20 and 50 × 50 km UTM cell local breed sampling, for the past and present distributions. Also, we performed a sensitivity test, removing those extinct and new recognised local livestock breeds for the analysis. Secondly, to account for the spatial heterogeneity and non-stationarity of the environmental gradients, we performed the GWR models varying the adaptive bandwidth, considering also 2.5%, 10%, 20% (i.e. ca. 50, 200 and 400 km bandwidth respectively)^83^. We chose an adaptive bandwidth, in order to facilitate result comparison within and between models.

We evaluated model accuracy using global quasi-R^2^ to assess the global explained variance to compare past and present richness distribution model performances. The global quasi-R^2^ is calculated from the coefficients in the local models, not by aggregating the local R^2^^2,84^. Lastly, we documented the spatial variation in regression coefficients and their statistical significance (at [*P* = 0] ≤ 0.05) to map only significant results and quantify their ratio.

Finally, to analyse the relations between land used changes and local livestock breeds, we performed Ordinal Logistic Regression (OLR) models^85^, where land cover intensification transition gradient was the response variable and local breed richness indices were the predictors. This technique has been proposed to analyses land uses changes, as it assumes ordinality of the outcomes and it is favourable when land cover change patterns can be interpreted as an ordinal process^86^ -in our case, ordered sequence of change in land cover types from extensification to intensification-. Lastly, we calculated the predicted probabilities for each of the levels of the predictors -i.e. breed richness by municipality.

All data processing and analyses were performed in R v3.6.0 software^68^ using the “sf”^87^ and “tidyverse”^88^ to process local livestock breed data, “raster” package^89^ to process the environmental data, “spgwr” package^90^ to perform GWR, “MASS”^91^ package to perform OLR and “sf”^87^ and “ggplot2”^92^ for result visualization.

## Supplementary information

Supplementary Information 1.

## Data Availability

The data that support the findings of this study will be openly available in a public repository. We are working on the elaboration of a data paper related to the livestock distribution information.
