# Supplementary material for: Recent land use and management changes decouple the adaptation of livestock diversity to the environment
Source: Sci Rep. 2020 Dec 3;10:21035. doi: 10.1038/s41598-020-77878-2 (PMC7713044; doi:10.1038/s41598-020-77878-2)
Supplement: Supplementary file 1 — Supplementary Information 1. [file 41598_2020_77878_MOESM1_ESM.docx]

**Recent land use and management changes decouple the adaptation of livestock diversity to the environment.**

Elena Velado-Alonso * ^a,b,1)^, Ignacio Morales-Castilla^b,c, 2)^, Antonio Gómez-Sal^a, b, 3)^

^a^ FORECO – Forest Ecology and Restoration Group, Department of Life Sciences, Universidad de Alcalá, Ctra. Madrid-Barcelona Km 33.600, 28805, Alcalá de Henares, Madrid, Spain.

^b^ GloCEE - Global Change Ecology and Evolution Group, Department of Life Sciences, Universidad de Alcalá, Ctra. Madrid-Barcelona Km 33.600, 28805, Alcalá de Henares, Madrid, Spain.

^c^ Department of Environmental Science and Policy, David King Hall Rm 3005, George Mason University, 4400 University Drive, Fairfax, VA 22030-4444, Virginia, USA.

^1)^ Corresponding author: 10000-0003-4805-2929. [elena.velado@uah.es](mailto:elena.velado@uah.es) & [velado.elena@gmail.com](mailto:velado.elena@gmail.com)

^2)^ 20000-0002-8570-9312. [ignacio.moralesc@uah.es](mailto:ignacio.moralesc@uah.es)

^3)^ 0000-0003-1925-4580. [antonio.gomez@uah.es](mailto:antonio.gomez@uah.es)

**Supplementary Information**

**Appendix 1. Local Livestock Breed Data**

Table S1. List of local, i.e. autochthonous, livestock breeds included in this work.

| **Breed Name** | **Livestock Type** | **Status** |
| --- | --- | --- |
| Asturiana de los Valles | Bovine | Increasing |
| Avileña Negra Ibérica | Bovine | Increasing |
| Lidia | Bovine | Increasing |
| Morucha | Bovine | Increasing |
| Parda de Montaña | Bovine | Increasing |
| Pirenaica | Bovine | Increasing |
| Retinta | Bovine | Increasing |
| Rubia Gallega | Bovine | Increasing |
| Albera | Bovine | Danger of Extinction |
| Alistano-Sanabresa | Bovine | Danger of Extinction |
| Asturiana de Montaña | Bovine | Danger of Extinction |
| Avileña Negra Ibérica Bociblanca | Bovine | Danger of Extinction |
| Berrenda en Colorado | Bovine | Danger of Extinction |
| Berrenda en Negro | Bovine | Danger of Extinction |
| Betizu | Bovine | Danger of Extinction |
| Blanca Cacereña | Bovine | Danger of Extinction |
| Bruna de los Pirineos | Bovine | Danger of Extinction |
| Cachena | Bovine | Danger of Extinction |
| Caldelá | Bovine | Danger of Extinction |
| Cárdena Andaluza | Bovine | Danger of Extinction |
| Frieiresa | Bovine | Danger of Extinction |
| Limia | Bovine | Danger of Extinction |
| Marismeña | Bovine | Danger of Extinction |
| Monchina | Bovine | Danger of Extinction |
| Morucha Negra | Bovine | Danger of Extinction |
| Murciano-Levantina | Bovine | Danger of Extinction |
| Negra Andaluza | Bovine | Danger of Extinction |
| Pajuna | Bovine | Danger of Extinction |
| Pallaresa | Bovine | Danger of Estinction |
| Pasiega | Bovine | Danger of Extinction |
| Sayaguesa | Bovine | Danger of Extinction |
| Serrana Negra | Bovine | Danger of Extinction |
| Serrana de Teruel | Bovine | Danger of Extinction |
| Terreña | Bovine | Danger of Extinction |
| Tudanca | Bovine | Danger of Extinction |
| Vianesa | Bovine | Danger of Extinction |
| Berciana | Bovine | Extinct |
| Campoo | Bovine | Extinct |
| Colorada Extremeña | Bovine | Extinct |
| Lebaniega | Bovine | Extinct |
| Mantequera Leonesa | Bovine | Extinct |
| Rubia Andaluza | Bovine | Extinct |
| Vaca del Pirineo Catalán | Bovine | Extinct |
| Verinesa | Bovine | Extinct |
| Castellana | Ovine | Increasing |
| Churra | Ovine | Increasing |
| Latxa | Ovine | Increasing |
| Manchega | Ovine | Increasing |
| Merino | Ovine | Increasing |
| Navarra | Ovine | Increasing |
| Ojinegra de Teruel | Ovine | Increasing |
| Rasa Aragonesa | Ovine | Increasing |
| Segureña | Ovine | Increasing |
| Alcarreña | Ovine | Danger of Extinction |
| Ansotana | Ovine | Danger of Extinction |
| Aranesa | Ovine | Danger of Extinction |
| Carranzana | Ovine | Danger of Extinction |
| Carranzana Negra | Ovine | Danger of Extinction |
| Cartera | Ovine | Danger of Extinction |
| Castellana Negra | Ovine | Danger of Extinction |
| Chamarita | Ovine | Danger of Extinction |
| Churra Lebrijana | Ovine | Danger of Extinction |
| Churra Tensina | Ovine | Danger of Extinction |
| Colmenareña | Ovine | Danger of Extinction |
| Galega | Ovine | Danger of Extinction |
| Guirra | Ovine | Danger of Extinction |
| Lojeña | Ovine | Danger of Extinction |
| Maellana | Ovine | Danger of Extinction |
| Manchega Negra | Ovine | Danger of Extinction |
| Mererino de Grazalema | Ovine | Danger of Extinction |
| Merina Negra | Ovine | Danger of Extinction |
| Merinno de los Montes Universales | Ovine | Danger of Extinction |
| Montesina | Ovine | Danger of Extinction |
| Ojalada | Ovine | Danger of Extinction |
| Ripollesa | Ovine | Danger of Extinction |
| Roya Bilbilitana | Ovine | Danger of Extinction |
| Rubia del Molar | Ovine | Danger of Extinction |
| Sasi Ardi | Ovine | Danger of Extinction |
| Talaverana | Ovine | Danger of Extinction |
| Xalda | Ovine | Danger of Extinction |
| Xisqueta | Ovine | Danger of Extinction |
| Blanca del Bierzo | Ovine | Extinct |
| Florida | Caprine | Increasing |
| Malagueña | Caprine | Increasing |
| Murciano-Granadina | Caprine | Increasing |
| Agrupación de las Mesetas | Caprine | Danger of Extinction |
| Azpi Gorri | Caprine | Danger of Extinction |
| Bermeya | Caprine | Danger of Extinction |
| Blanca Andaluza | Caprine | Danger of Extinction |
| Blanca Celtibérica | Caprine | Danger of Extinction |
| Blanca de Rasquera | Caprine | Danger of Extinction |
| Galega | Caprine | Danger of Extinction |
| (Cabra del) Guadarrama | Caprine | Danger of Extinction |
| Moncaina | Caprine | Danger of Extinction |
| Negra Serrana | Caprine | Danger of Extinction |
| Payoya | Caprine | Danger of Extinction |
| Pirenaica | Caprine | Danger of Extinction |
| Retinta | Caprine | Danger of Extinction |
| Verata | Caprine | Danger of Extinction |
| Guisandesa | Caprine | Extinct |
| Jurdana | Caprine | Extinct |
| Española | Equid | Increasing |
| Asno Andaluz | Equid (Donkey) | Danger of Extinction |
| Asno de las Encartaciones | Equid (Donkey) | Danger of Extinction |
| Asno Catalán | Equid (Donkey) | Danger of Extinction |
| Asno Zamorano-Leonés | Equid (Donkey) | Danger of Extinction |
| Caballo Asturcón | Equid | Danger of Extinction |
| Caballo de Burguete | Equid | Danger of Extinction |
| Caballo Hispano-Árabe | Equid | Danger of Extinction |
| Caballo Hispano Bretón | Equid | Danger of Extinction |
| Caballo Losino | Equid | Danger of Extinction |
| Caballo Marismeño | Equid | Danger of Extinction |
| Caballo de Monte del País Vasco | Equid | Danger of Extinction |
| Caballo Monchino | Equid | Danger of Extinction |
| Jaca Navarra | Equid | Danger of Extinction |
| Cavall del Pirenenc Català | Equid | Danger of Extinction |
| Caballo Pottoka | Equid | Danger of Extinction |
| Cabalo Pura Raza Galega | Equid | Danger of Extinction |
| Caballo de las Retuertas | Equid | Danger of Extinction |
| Ibérico | Porcine | Increasing |
| Ibérico Entrepelado (*new breed*) | Porcine | Increasing |
| Ibérico Retinto (*new breed*) | Porcine | Increasing |
| Ibérico Lampiño (*new breed*) | Porcine | Danger of Extinction |
| Ibérico Manchado de Jabugo (*new breed*) | Porcine | Danger of Extinction |
| Ibérico Torbiscal (*new breed*) | Porcine | Danger of Extinction |
| Celta | Porcine | Danger of Extinction |
| Chato Murciano | Porcine | Danger of Extinction |
| Euskal Txerria | Porcine | Danger of Extinction |
| Gochu Asturcelta | Porcine | Danger of Extinction |
| Baztanesa | Porcine | Extinct |
| Cerdo de Vich | Porcine | Extinct |
| Chato Vitoriano | Porcine | Extinct |
| Lermeña | Porcine | Extinct |

Table S2. List of bibliographic sources reviewed for Spanish livestock autochthonous breed areas of origin identification.

| Authors | Year | Edition | Title |
| --- | --- | --- | --- |
| Faelli, F. | 1932 | Edición Española- Revista Veterinaria de España (1ªEdición 1902) | Razas Bovinas, Equinas, Porcinas, Ovinas y Caprinas. Traducción anotada por Tomás de la Fuente Muñoz. |
| Aparicio, G. | 1947 | Segunda Edición (1ª Edición 1944) | Zootecnia Especial. Etnología compendiada. |
| Esteban Muñoz, C. & Tejón Tejón, D. | 1980 | Primera Edición | Catálogo de Razas Autóctonas Españolas. I-Especies Ovina y Caprina |
| Sánchez Belda, A., | 1984 | Primera Edición | Razas Bovinas Españolas |
| Sámchez Belda, A. & Sánchez Trujillano, M.C. | 1986 | Segunda Edición (Primera Edición 1974). | Razas Ovinas Españolas |
| García Dory, M.A., Martínez Vicente, S. & Orozco Piñán, F. | 1990 | Primera Edición | Guía de Campo de las Razas Autóctonas de España |
| Sáncehz Belda, A. | 2002 | Primera Edición | Razas Ganaderas Españolas Bovinas |
| Esteban Muñoz, C. | 2003 | Primera Edición | Razas Ganaderas Españolas Ovinas |
| Yanes García, J.E. | 2005 | Primera Edición | Razas Asnales Autóctonas Españolas |
| Esteban Muñoz, C. | 2008 | Primera Edición | Razas Ganaderas Españolas Caprinas |
| Sánchez Belda, A. | 2012 | Primera Edición | Razas Ganaderas Españolas Caballares |
| Information available on the ARCA (Breed Information National System) website of the MAPA (Ministry of Agriculture, Fisheries and Food) has also been consulted: <http://www.mapama.gob.es/es/ganaderia/temas/zootecnia/razas-ganaderas/razas/catalogo/>  consulted between March and July, 2017 | | | |

Table S3. Percentage of farms included in this study for those increasing in number local, i.e. autochthonous, breeds obtained from the comparison between collaborating farms on the national conservation program and active farms on the Official Breeding Books. For four breeds that comparison was not possible, due to that we are not sure about the included percentage, however we consider that probably is less than 20%, nonetheless that is the information facilitated by the

| **Breed Name (species)** | **Farm percentage** |
| --- | --- |
| *Asturiana de los Valles (Bovine)* | 100% |
| *Castellana (Ovine)* | 100% |
| *Churra (Ovine)* | 100% |
| *Navarra (Ovine)* | 100% |
| *Rubia Gallega (Bovine)* | 100% |
| *Merina (Ovine)* | 89% |
| *Marchega (Ovine)* | 86% |
| *Murciano-Granadina (Caprine)* | 85% |
| *Latxa (Ovine)* | 83% |
| *Lidia (Bovine)* | 81% |
| *Rasa Aragonesa (Ovine)* | 77% |
| *Pirenaica (Bovine)* | 73% |
| *Retinta (Bovine)* | 68% |
| *Parda de Montaña (Bovine)* | 65% |
| *Florida (Caprine)* | 60% |
| *Segureña (Ovine)* | 50% |
| *Avileña-Negra Ibérica (Bovine)* | 50% |
| *Ojinegra de Teruel* | 34% |
| *Pirenaica (Bovine)* | 30% |
| *Carranzana (Ovine)* | 24% |
| *Morucha (Bovine)* | 21% |
| *Española (Equid)* | Unsure |
| *Ibérico (Porcine)* | Unsure |
| *Ibérico Entrepelado (Porcine)* | Unsure |
| *Ibérico Retinto (Porcine)* | Unsure |

**Appendix 2. Geographic Weighted Regression Models**

1. **Sampling Effects**

Table S1. Quasi-global R^2^ Geographically Weighted Regression (analyses extent: 5% of data) fitted models performed for 3 different sampling scales (10×10, 20×20, 50x50 km UTM grid cell) of the autochthonous breed richness (bovine, ovine, caprine, equid -hoses and donkeys-, porcine species and total) for past and present distribution, using as predictors annual mean temperature, annual precipitation, precipitation seasonality and vegetation productivity seasonality.

|  | **UTM** | **Past** | **Present** |
| --- | --- | --- | --- |
|  |  | **Quasi- Global R^2^** | **Quasi- Global R^2^** |
| **Bovine** | *10x10* | 0.63 | 0.45 |
|  | *20X20* | 0.65 | 0.37 |
|  | *50x50* | 0.66 | 0.48 |
| **Ovine** | *10x10* | 0.39 | 0.20 |
|  | *20x20* | 0.43 | 0.16 |
|  | *50x50* | 0.57 | 0.24 |
| **Caprine** | *10x10* | 0.40 | 0.29 |
|  | *20x20* | 0.43 | 0.23 |
|  | *50x50* | 0.46 | 0.35 |
| **Equid** | *10x10* | 0.73 | 0.41 |
|  | *20x20* | 0.73 | 0.42 |
|  | *50x50* | 0.75 | 0.47 |
| **Porcine** | *10x10* | 0.66 | 0.35 |
|  | *20x20* | 0.66 | 0.24 |
|  | *50x50* | 0.70 | 0.38 |
| **Total** | *10x10* | 0.64 | 0.46 |
|  | *20x20* | 0.65 | 0.41 |
|  | *50x50* | 0.68 | 0.51 |

Table S2. Summary of GWR coefficient estimates *(β)* for past distribution of livestock autochthonous breed richness (total, bovine, ovine, caprine, equid and porcine) sampled at 20 × 20 km UTM cell, using 5% of the data as bandwidth.

| **Past** | **20x20 UTM grid Cell** | **Min.** | **1st Q.** | **Median** | **3rd Q.** | **Max.** | **Global** |
| --- | --- | --- | --- | --- | --- | --- | --- |
| **Total Breeds** | *Intercept* | 0.2101 | 2.614 | 3.42 | 4.256 | 13.229 | 4.923 |
|  | *Annual Mean Temperature* | -3.609 | -2.182 | -1.391 | -0.73 | 3.536 | -1.273 |
|  | *Annual Precipitation* | -4.151 | -1.202 | -0.226 | 0.894 | 8.606 | -0.413 |
|  | *Precipitation Seasonality* | -4.217 | -0.819 | 0.89 | 2.919 | 6.307 | 2.036 |
|  | *Seasonality of Vegetation Productivity* | -1.105 | -0.102 | 0.152 | 0.426 | 1.312 | 0.903 |
| **Bovine** | *Intercept* | -0.895 | 0.306 | 0.918 | 1.477 | 4.898 | 1.52 |
|  | *Annual Mean Temperature* | -1.664 | -0.841 | -0.391 | -0.056 | 2.34 | -0.438 |
|  | *Annual Precipitation* | -1.839 | -0.331 | 0.145 | 0.51 | 4.06 | -0.162 |
|  | *Precipitation Seasonality* | -2.136 | -0.694 | 0.22 | 0.985 | 2.385 | 0.796 |
|  | *Seasonality of Vegetation Productivity* | -0.247 | -0.025 | 0.096 | 0.201 | 0.753 | 0.478 |
| **Ovine** | *Intercept* | 0.163 | 1.147 | 1.503 | 1.928 | 4.478 | 1.503 |
|  | *Annual Mean Temperature* | -1.846 | -0.84 | -0.66 | -0.511 | 1.1129 | -0.571 |
|  | *Annual Precipitation* | -2.757 | -0.66 | -0.412 | -0.188 | 2.811 | -0.423 |
|  | *Precipitation Seasonality* | -2.076 | 0.017 | 0.338 | 0.676 | 2.519 | 0.413 |
|  | *Seasonality of Vegetation Productivity* | -0.263 | -0.143 | 0.02 | 0.244 | 0.527 | 0.079 |
| **Caprine** | *Intercept* | 0.0.67 | 0.434 | 0.72 | 0.937 | 1.778 | 0.655 |
|  | *Annual Mean Temperature* | -0.819 | -0.549 | -0.425 | -0.323 | 0.624 | -0.498 |
|  | *Annual Precipitation* | -0.783 | -0.244 | -0.054 | 0.135 | 1.232 | -0.196 |
|  | *Precipitation Seasonality* | -0.625 | -0.087 | 0.19 | 0.416 | 1.03 | 0.319 |
|  | *Seasonality of Vegetation Productivity* | -0.451 | -0.169 | -0.105 | 0.002 | 0.225 | -0.007 |
| **Equid** | *Intercept* | -2.598 | -0.234 | 0.172 | 0.338 | 0.93 | 0.779 |
|  | *Annual Mean Temperature* | -1.215 | -0.405 | -0.139 | 0.161 | 0.409 | 0.058 |
|  | *Annual Precipitation* | -3.228 | -1.212 | 0.123 | 0.444 | 0.904 | 0.14 |
|  | *Precipitation Seasonality* | -0.91 | -0.444 | -0.13 | 1.584 | 2.686 | 0.359 |
|  | *Seasonality of Vegetation Productivity* | -0.454 | -0.134 | 0.018 | 0.096 | 0.279 | 0.195 |
| **Porcine** | *Intercept* | -0.198 | 0.19 | 0.386 | 0.619 | 1.827 | 0.466 |
|  | *Annual Mean Temperature* | -0.201 | 0.056 | 0.216 | 0.329 | 0.873 | 0.176 |
|  | *Annual Precipitation* | -0.645 | 0.106 | 0.277 | 0.393 | 1.975 | 0.229 |
|  | *Precipitation Seasonality* | -0.941 | -0.153 | -0.021 | 0.179 | 0.67 | 0.149 |
|  | *Seasonality of Vegetation Productivity* | -0.175 | -0.002 | 0.066 | 0.191 | 0.32 | 0.157 |

Table S3. Summary of GWR coefficient estimates *(β)* for present distribution of livestock autochthonous breed richness (total, bovine, ovine, caprine, equid and porcine) sampled at 20 × 20 km UTM cell, using 5% of the data as bandwidth.

| **Present** | **20x20 UTM grid Cell** | **Min.** | **1st Q.** | **Median** | **3rd Q.** | **Max.** | **Global** |
| --- | --- | --- | --- | --- | --- | --- | --- |
| **Total Breeds** | *Intercept* | 0.606 | 1.436 | 1.987 | 2.606 | 7.0295 | 2.488 |
|  | *Annual Mean Temperature* | -1.166 | -0.051 | 0.615 | 1.318 | 5.228 | 0.632 |
|  | *Annual Precipitation* | -2.584 | -0.141 | 0.521 | 1.37 | 6.136 | 0.403 |
|  | *Precipitation Seasonality* | -4.31 | -0.965 | -0.056 | 0.784 | 2.875 | 0.622 |
|  | *Seasonality of Vegetation Productivity* | 0.008 | 0.271 | 0.634 | 0.9801 | 1.922 | 0.873 |
| **Bovine** | *Intercept* | -0.177 | 0.429 | 0.798 | 1.301 | 3.3467 | 0.856 |
|  | *Annual Mean Temperature* | -0.539 | -0.016 | 0.154 | 0.544 | 2.401 | 0.183 |
|  | *Annual Precipitation* | -0.383 | 0.07 | 0.395 | 0.841 | 3.892 | 0.27 |
|  | *Precipitation Seasonality* | -2.169 | -0.58 | -0.149 | 0.218 | 1.093 | 0.209 |
|  | *Seasonality of Vegetation Productivity* | -0.015 | 0.108 | 0.297 | 0.538 | 1.082 | 0.416 |
| **Ovine** | *Intercept* | 0.036 | 0.262 | 0.409 | 0.564 | 1.611 | 0.384 |
|  | *Annual Mean Temperature* | -0.295 | -0.045 | 0.134 | 0.205 | 0.758 | 0.061 |
|  | *Annual Precipitation* | -0.754 | -0.219 | 0.032 | 0.15 | 1.12 | -0.001 |
|  | *Precipitation Seasonality* | -1.016 | -0.247 | -0.079 | 0.052 | 0.411 | -0.046 |
|  | *Seasonality of Vegetation Productivity* | -0.161 | 0.018 | 0.066 | 0.177 | 0.362 | 0.082 |
| **Caprine** | *Intercept* | -0.444 | 0.03 | 0.118 | 0.226 | 0.696 | 0.234 |
|  | *Annual Mean Temperature* | -0.475 | -0.156 | -0.0136 | 0.045 | 0.32 | 0.016 |
|  | *Annual Precipitation* | -1.363 | -0.171 | 0.084 | 0.168 | 0.549 | 0.025 |
|  | *Precipitation Seasonality* | -0.372 | -0.022 | 0.061 | 0.258 | 0.65 | 0.08 |
|  | *Seasonality of Vegetation Productivity* | -0.149 | 0.024 | 0.026 | 0.067 | 187 | 0.025 |
| **Equid** | *Intercept* | -0.57 | 0.424 | 0.583 | 0.739 | 1.327 | 0.839 |
|  | *Annual Mean Temperature* | -0.377 | 0.097 | 0.251 | 0.48 | 1.478 | 0.294 |
|  | *Annual Precipitation* | -1.651 | -0.371 | 0.088 | 0.28 | 0.56 | 0.07 |
|  | *Precipitation Seasonality* | -0.94 | -0.013 | 0.22 | 0.413 | 1.162 | 0.252 |
|  | *Seasonality of Vegetation Productivity* | -0.04 | 0.088 | 0.175 | 0.235 | 0.462 | 0.27 |
| **Porcine** | *Intercept* | -0.178 | 0.007 | 0.066 | 0.192 | 2.127 | 0.175 |
|  | *Annual Mean Temperature* | -0.265 | 0 | 0.025 | 0.175 | 0.691 | 0.078 |
|  | *Annual Precipitation* | -0.616 | -0.001 | 0.009 | 0.572 | 1.56 | 0.039 |
|  | *Precipitation Seasonality* | -0.952 | -0.178 | 0.001 | 0.064 | 0.882 | 0.127 |
|  | *Seasonality of Vegetation Productivity* | -0.01 | 0.002 | 0.029 | 0.071 | 0.192 | 0.079 |

Table S4. Summary of GWR coefficient estimates *(β)* for past distribution of livestock autochthonous breed richness (total, bovine, ovine, caprine, equid and porcine) sampled at 50 × 50 km UTM cell, using 5% of the data as bandwidth.

| **Past** | **50x50 UTM grid Cell** | **Min.** | **1st Q.** | **Median** | **3rd Q.** | **Max.** | **Global** |
| --- | --- | --- | --- | --- | --- | --- | --- |
| **Total Breeds** | *Intercept* | -1.377 | 3.369 | 4.188 | 5.27 | 15.503 | 6.278 |
|  | *Annual Mean Temperature* | -6.011 | -3.587 | -2.382 | -1.377 | 2.708 | -1.757 |
|  | *Annual Precipitation* | -6.823 | -2.553 | -0.833 | 0.018 | 7.576 | 0.773 |
|  | *Precipitation Seasonality* | -4.903 | -0.708 | 1.066 | 3.968 | 8.645 | 2.239 |
|  | *Seasonality of Vegetation Productivity* | -1.011 | -0.056 | 0.537 | 1.16 | 2.033 | 1.374 |
| **Bovine** | *Intercept* | -1.298 | 0.619 | 1.221 | 1.833 | 6.048 | 2 |
|  | *Annual Mean Temperature* | -2.664 | -1.337 | -0.671 | -0.29 | 2.014 | -0.606 |
|  | *Annual Precipitation* | -2.602 | -0.95 | -0.261 | 0.135 | 3.163 | -0.251 |
|  | *Precipitation Seasonality* | -2.547 | -0.455 | 0.646 | 1.454 | 3.116 | 0.988 |
|  | *Seasonality of Vegetation Productivity* | -0.5 | 0.081 | 0.299 | 0.532 | 1.253 | 0.719 |
| **Ovine** | *Intercept* | 0.363 | 1.221 | 1.584 | 2.227 | 5.141 | 1.932 |
|  | *Annual Mean Temperature* | -2.436 | -1.291 | -1.076 | -0.845 | 0.275 | -0.859 |
|  | *Annual Precipitation* | -2.859 | -1.52 | -0.516 | -0.313 | 3.062 | -0.605 |
|  | *Precipitation Seasonality* | -1.605 | -0.002 | 0.357 | 0.924 | 2.715 | 0.46 |
|  | *Seasonality of Vegetation Productivity* | -0.548 | -0.127 | 0.066 | 0.259 | 0.739 | 0.064 |
| **Caprine** | *Intercept* | 0.32 | 0.663 | 0.812 | 1.073 | 2.957 | 0.858 |
|  | *Annual Mean Temperature* | -1.433 | -0.804 | -0.588 | -0.439 | 0.062 | -0.571 |
|  | *Annual Precipitation* | -1.077 | -0.367 | -0.192 | -0.051 | 1.846 | -0.258 |
|  | *Precipitation Seasonality* | -0.494 | 0.038 | 0.293 | 0.496 | 1.202 | 0.382 |
|  | *Seasonality of Vegetation Productivity* | -0.549 | -0.181 | -0.079 | 0.046 | 0.256 | 0.017 |
| **Equid** | *Intercept* | -2.139 | -0.13 | 0.264 | 0.468 | 1.546 | 0.919 |
|  | *Annual Mean Temperature* | -1.355 | -0.529 | -0.069 | 0.279 | 1.078 | 0.104 |
|  | *Annual Precipitation* | -2.545 | -0.954 | 0.097 | 0.37 | 0.974 | 0.133 |
|  | *Precipitation Seasonality* | -1.663 | -0.608 | -0.297 | 1.321 | 3.078 | 0.292 |
|  | *Seasonality of Vegetation Productivity* | -0.566 | -0.149 | 0.026 | 0.249 | 0.826 | 0.34 |
| **Porcine** | *Intercept* | -0.49 | 0.209 | 0.542 | 0.805 | 1.509 | 0.57 |
|  | *Annual Mean Temperature* | -0.342 | 0.0753 | 0.185 | 0.318 | 0.68 | 0.176 |
|  | *Annual Precipitation* | -0.99 | 0.11 | 0.189 | 0.286 | 1.309 | 0.208 |
|  | *Precipitation Seasonality* | -0.713 | -0.179 | -0.013 | 0.18 | 0.684 | 0.116 |
|  | *Seasonality of Vegetation Productivity* | -0.087 | 0.014 | 0.088 | 0.284 | 0.435 | 0.235 |

Table S5. Summary of GWR coefficient estimates *(β)* for present distribution of livestock autochthonous breed richness (total, bovine, ovine, caprine, equid and porcine) sampled at 50 × 50 km UTM cell, using 5% of the data as bandwidth.

| **Present** | **50x50 UTM grid Cell** | **Min.** | **1st Q.** | **Median** | **3rd Q.** | **Max.** | **Global** |
| --- | --- | --- | --- | --- | --- | --- | --- |
| **Total Breeds** | *Intercept* | -0.31 | 1.744 | 2.409 | 3.189 | 10.308 | 3.081 |
|  | *Annual Mean Temperature* | -0.654 | 0.116 | 0.946 | 1.853 | 4.104 | 0.875 |
|  | *Annual Precipitation* | -3.094 | -0.133 | 0.584 | 1.318 | 8.553 | 0.534 |
|  | *Precipitation Seasonality* | -4.421 | -1.489 | -0.519 | 0.648 | 3.49 | 0.622 |
|  | *Seasonality of Vegetation Productivity* | -0.151 | 0.303 | 0.641 | 1.156 | 2.437 | 1.021 |
| **Bovine** | *Intercept* | -0.39 | 0.512 | 0.882 | 1.802 | 4.763 | 1.064 |
|  | *Annual Mean Temperature* | -0.249 | 0.138 | 0.369 | 1.077 | 3.753 | 0.357 |
|  | *Annual Precipitation* | -1.222 | 0.212 | 0.515 | 1.21 | 5.623 | 0.347 |
|  | *Precipitation Seasonality* | -3.306 | -0.954 | -0.37 | 0.007 | 1.653 | 0.182 |
|  | *Seasonality of Vegetation Productivity* | -0.307 | 0.034 | 0.184 | 0.556 | 1.51 | 0.47 |
| **Ovine** | *Intercept* | -0.43 | 0.162 | 0.43303 | 0.67 | 2.131 | 0.458 |
|  | *Annual Mean Temperature* | -0.759 | -0.067 | 0.049 | 0.128 | 0.589 | 0.056 |
|  | *Annual Precipitation* | -1.557 | -0.465 | -0.048 | 0.048 | 1.163 | -0.06 |
|  | *Precipitation Seasonality* | -0.928 | -0.5 | -0.147 | 0.02 | 0.674 | -0.068 |
|  | *Seasonality of Vegetation Productivity* | -0.463 | 0.036 | 0.129 | 0.197 | 0.474 | 0.121 |
| **Caprine** | *Intercept* | -0.578 | 0.06 | 0.142 | 0.392 | 0.925 | 0.285 |
|  | *Annual Mean Temperature* | -0.766 | -0.289 | -0.092 | 0.008 | 0.247 | -0.023 |
|  | *Annual Precipitation* | -1.584 | -0.209 | 0.107 | 0.217 | 0.676 | 0.047 |
|  | *Precipitation Seasonality* | -0.384 | 0.073 | 0.244 | 0.364 | 0.863 | 0.107 |
|  | *Seasonality of Vegetation Productivity* | -0.415 | -0.043 | 0.035 | 0.148 | 0.41 | 0.005 |
| **Equid** | *Intercept* | -0.276 | 0.517 | 0.889 | 1.09 | 2.855 | 1.048 |
|  | *Annual Mean Temperature* | -0.266 | 0.097 | 0.3 | 0.553 | 0.823 | 0.355 |
|  | *Annual Precipitation* | -1.149 | -0.24 | 0.156 | 0.385 | 1.005 | 0.115 |
|  | *Precipitation Seasonality* | -1.148 | -0.277 | 0.13 | 0.505 | 1.064 | 0.25 |
|  | *Seasonality of Vegetation Productivity* | -0.164 | 0.077 | 0.24 | 0.355 | 0.531 | 0.299 |
| **Porcine** | *Intercept* | -0.502 | 0 | 0.009 | 0.025 | 1.795 | 0.227 |
|  | *Annual Mean Temperature* | -0.731 | 0 | 0.026 | 0.241 | 0.751 | 0.129 |
|  | *Annual Precipitation* | -1.284 | 0 | 0.031 | 0.205 | 1.399 | 0.085 |
|  | *Precipitation Seasonality* | -0.827 | -0.161 | 0.038 | 0.034 | 2.021 | 0.152 |
|  | *Seasonality of Vegetation Productivity* | -0.234 | 0 | 0.018 | 0.102 | 0.467 | 0.126 |

1. **Analysis Extent Effects**

Table S6: Quasi-global R^2^ from the Geographically Weighted Regression fitted models performed at 4 analysis extent (2.5%, 5%, 10%, 20% of the total data as bandwidth) of the local, i.e. autochthonous, breed richness (bovine, ovine, caprine, equid -hoses and donkeys-, porcine species and total, sampled at 10×10 km UTM grid cell) for past and present distributions, using as predictors annual mean temperature, annual precipitation, precipitation seasonality and vegetation productivity seasonality.

| **Sampling size:**  **10×10 UTM cell** | **Analysis Extent**  **(data %)** | **Quasi- Global R^2^** | |
| --- | --- | --- | --- |
|  |  | **Past** | **Present** |
| **Bovine** | *2.50%* | 0.71 | 0.52 |
|  | *5%* | 0.63 | 0.45 |
|  | *10%* | 0.54 | 0.38 |
|  | *20%* | 0.45 | 0.31 |
| **Ovine** | *2.50%* | 0.54 | 0.28 |
|  | *5%* | 0.39 | 0.2 |
|  | *10%* | 0.26 | 0.14 |
|  | *20%* | 0.18 | 0.08 |
| **Caprine** | *2.50%* | 0.51 | 0.35 |
|  | *5%* | 0.4 | 0.29 |
|  | *10%* | 0.31 | 0.23 |
|  | *20%* | 0.26 | 0.17 |
| **Equid** | *2.50%* | 0.8 | 0.46 |
|  | *5%* | 0.73 | 0.41 |
|  | *10%* | 0.65 | 0.35 |
|  | *20%* | 0.54 | 0.31 |
| **Porcine** | *2.50%* | 0.72 | 0.43 |
|  | *5%* | 0.66 | 0.35 |
|  | *10%* | 0.6 | 0.28 |
|  | *20%* | 0.54 | 0.22 |
| **Total** | *2.50%* | 0.73 | 0.53 |
|  | *5%* | 0.64 | 0.46 |
|  | *10%* | 0.55 | 0.4 |
|  | *20%* | 0.46 | 0.35 |

Table S7. Summary of GWR coefficient estimates *(β)* for past distribution of livestock autochthonous breed richness (total, bovine, ovine, caprine, equid and porcine) sampled at 10 × 10 km UTM cell, using 2.5% of the data as bandwidth.

| **Past** | **Bandwidth = 2.5%** | **Min.** | **1st Q.** | **Median** | **3rd Q.** | **Max.** | **Global** |
| --- | --- | --- | --- | --- | --- | --- | --- |
| **Total Breeds** | *Intercept* | -4.18 | 1.38 | 2.40 | 4.18 | 4.18 | 4.36 |
|  | *Annual Mean Temperature* | 4.68 | -1.52 | -1.03 | -0.48 | 0.48 | -0.98 |
|  | *Annual Precipitation* | -10.01 | -0.90 | 0.15 | 1.13 | 1.14 | 1.84 |
|  | *Precipitation Seasonality* | 9.58 | -1.44 | 0.00 | 2.13 | 2.13 | 1.84 |
|  | *Seasonality of Vegetation Productivity* | -0.92 | -0.22 | 0.07 | 0.40 | 0.40 | 0.76 |
| **Bovine** | *Intercept* | -1.62 | 0.06 | 0.67 | 1.41 | 6.97 | 1.32 |
|  | *Annual Mean Temperature* | -1.92 | -0.73 | -0.36 | 0.12 | 3.16 | -0.34 |
|  | *Annual Precipitation* | -3.80 | -0.46 | 0.23 | 0.79 | 5.90 | -0.14 |
|  | *Precipitation Seasonality* | -3.78 | -0.66 | -0.09 | 0.96 | 3.24 | 0.71 |
|  | *Seasonality of Vegetation Productivity* | -0.49 | -0.08 | 0.03 | 0.14 | 0.81 | 0.38 |
| **Ovine** | *Intercept* | -1.87 | 0.74 | 1.38 | 2.04 | 6.39 | 1.35 |
|  | *Annual Mean Temperature* | -2.93 | -0.64 | -0.38 | 0.00 | 2.46 | -0.43 |
|  | *Annual Precipitation* | -5.97 | -0.60 | -0.28 | 0.22 | 5.13 | -0.38 |
|  | *Precipitation Seasonality* | -3.83 | -0.45 | 0.11 | 0.72 | 4.29 | 0.35 |
|  | *Seasonality of Vegetation Productivity* | -0.30 | -0.12 | 0.02 | 0.21 | 0.67 | 0.09 |
| **Caprine** | *Intercept* | -1.49 | 0.17 | 0.70 | 0.93 | 2.56 | 0.56 |
|  | *Annual Mean Temperature* | -1.52 | -0.45 | -0.33 | -0.17 | 0.88 | -0.43 |
|  | *Annual Precipitation* | -2.96 | -0.27 | 0.00 | 0.33 | 2.49 | -0.17 |
|  | *Precipitation Seasonality* | -1.09 | -0.37 | -0.01 | 0.38 | 2.29 | 0.26 |
|  | *Seasonality of Vegetation Productivity* | -0.36 | -0.14 | -0.07 | 0.01 | 0.25 | 0.01 |
| **Equid** | *Intercept* | -3.75 | -0.45 | 0.02 | 0.24 | 2.85 | 0.70 |
|  | *Annual Mean Temperature* | -1.85 | -0.20 | -0.01 | 0.12 | 1.18 | 0.05 |
|  | *Annual Precipitation* | -4.66 | -0.65 | 0.04 | 0.36 | 1.20 | 0.13 |
|  | *Precipitation Seasonality* | -1.68 | -0.50 | -0.09 | 0.80 | 4.03 | 0.36 |
|  | *Seasonality of Vegetation Productivity* | -0.42 | -0.09 | 0.00 | 0.07 | 0.25 | 0.14 |
| **Porcine** | *Intercept* | -0.75 | 0.12 | 0.26 | 0.70 | 2.43 | 0.43 |
|  | *Annual Mean Temperature* | -0.43 | 0.03 | 0.14 | 0.30 | 1.20 | 0.17 |
|  | *Annual Precipitation* | -1.48 | 0.05 | 0.23 | 0.40 | 2.70 | 0.23 |
|  | *Precipitation Seasonality* | -1.42 | -0.23 | -0.02 | 0.10 | 1.04 | 0.16 |
|  | *Seasonality of Vegetation Productivity* | -0.16 | 0.00 | 0.03 | 0.12 | 0.33 | 0.14 |

Table S8. Summary of GWR coefficient estimates *(β)* for present distribution of livestock autochthonous breed richness (total, bovine, ovine, caprine, equid and porcine) sampled at 10 × 10 km UTM cell, using 2.5% of the data as bandwidth.

| **Present** | **Bandwidth = 2.5%** | **Min.** | **1st Q.** | **Median** | **3rd Q.** | **Max.** | **Global** |
| --- | --- | --- | --- | --- | --- | --- | --- |
| **Total Breeds** | *Intercept* | -0.06 | 2.07 | 3.66 | 5.99 | 15.91 | 4.66 |
|  | *Annual Mean Temperature* | -2.72 | -0.19 | 0.38 | 1.16 | 8.7 | 0.61 |
|  | *Annual Precipitation* | -6.07 | 0.12 | 1.13 | 2.57 | 10.34 | 0.88 |
|  | *Precipitation Seasonality* | -6.51 | -2.27 | -1.26 | 1.22 | 4.38 | 0.95 |
|  | *Seasonality of Vegetation Productivity* | -0.52 | 0.06 | 0.46 | 1.08 | 2.27 | 1.02 |
| **Bovine** | *Intercept* | -0.36 | 0.73 | 1.36 | 2.82 | 6.96 | 1.62 |
|  | *Annual Mean Temperature* | -1.69 | -0.38 | 0.05 | 0.48 | 4.42 | 0.03 |
|  | *Annual Precipitation* | -4.92 | 0.17 | 0.66 | 1.61 | 6.03 | 0.49 |
|  | *Precipitation Seasonality* | -4.4 | -0.9 | -0.28 | 0.48 | 3.16 | 0.49 |
|  | *Seasonality of Vegetation Productivity* | -0.4 | 0 | 0.2 | 0.57 | 1.38 | 0.49 |
| **Ovine** | *Intercept* | -0.21 | 0.41 | 0.8 | 1.26 | 3.51 | 0.78 |
|  | *Annual Mean Temperature* | -0.85 | -0.13 | 0.07 | 0.31 | 1.39 | 0.01 |
|  | *Annual Precipitation* | -1.82 | -0.28 | 0 | 0.57 | 3.33 | -0.02 |
|  | *Precipitation Seasonality* | -1.9 | -0.68 | -0.27 | 0.17 | 1.08 | -0.06 |
|  | *Seasonality of Vegetation Productivity* | -0.35 | -0.04 | 0.05 | 0.15 | 0.44 | 0.1 |
| **Caprine** | *Intercept* | -1.41 | 0.1 | 0.22 | 0.44 | 2.25 | 0.47 |
|  | *Annual Mean Temperature* | -1.68 | -0.29 | -0.01 | 0.1 | 1.33 | 0.01 |
|  | *Annual Precipitation* | -3.37 | -0.2 | 0.19 | 0.36 | 1.98 | 0.06 |
|  | *Precipitation Seasonality* | -1.4 | -0.15 | -0.01 | 0.27 | 1.64 | 0.19 |
|  | *Seasonality of Vegetation Productivity* | -0.25 | -0.08 | -0.02 | 0.08 | 0.38 | -0.01 |
| **Equid** | *Intercept* | -0.8 | 0.48 | 0.89 | 1.34 | 2.83 | 1.47 |
|  | *Annual Mean Temperature* | -1.09 | 0.03 | 0.39 | 0.6 | 1.17 | 0.45 |
|  | *Annual Precipitation* | -3.21 | -0.47 | 0.25 | 0.69 | 1.12 | 0.25 |
|  | *Precipitation Seasonality* | -2.64 | -0.72 | 0.07 | 0.51 | 2.36 | 0.08 |
|  | *Seasonality of Vegetation Productivity* | -0.15 | 0.07 | 0.16 | 0.28 | 0.49 | 0.32 |
| **Porcine** | *Intercept* | -0.47 | 0.02 | 0.12 | 0.35 | 4.62 | 0.31 |
|  | *Annual Mean Temperature* | -2.56 | -0.02 | 0.02 | 0.29 | 1.89 | 0.12 |
|  | *Annual Precipitation* | -2.46 | -0.05 | 0.03 | 0.3 | 4.33 | 0.1 |
|  | *Precipitation Seasonality* | -2.19 | -0.27 | 0.01 | 0.21 | 1.8 | 0.24 |
|  | *Seasonality of Vegetation Productivity* | -0.12 | 0 | 0.02 | 0.09 | 0.28 | 0.11 |

Table S9. Summary of GWR coefficient estimates *(β)* for past distribution of livestock autochthonous breed richness (total, bovine, ovine, caprine, equid and porcine) sampled at 10 × 10 km UTM cell, using 10% of the data as bandwidth.

| **Past** | **Bandwidth = 10%** | **Min.** | **1st Q.** | **Median** | **3rd Q.** | **Max.** | **Global** |
| --- | --- | --- | --- | --- | --- | --- | --- |
| **Total Breeds** | *Intercept* | 1.73 | 2.78 | 3.55 | 3.87 | 5.83 | 4.36 |
|  | *Annual Mean Temperature* | -2.44 | -1.31 | -0.96 | -0.71 | 1.41 | -0.98 |
|  | *Annual Precipitation* | -2.38 | -0.66 | 0.23 | 0.74 | 1.94 | -0.32 |
|  | *Precipitation Seasonality* | -1.45 | 0.11 | 1.4 | 2.65 | 4.64 | 1.84 |
|  | *Seasonality of Vegetation Productivity* | -0.36 | 0.04 | 0.27 | 0.39 | 0.7 | 0.76 |
| **Bovine** | *Intercept* | 0.12 | 0.78 | 1.08 | 1.31 | 2.46 | 1.32 |
|  | *Annual Mean Temperature* | -0.91 | -0.51 | -0.29 | 0.05 | 1.3 | -0.34 |
|  | *Annual Precipitation* | -0.66 | -0.09 | 0.39 | 0.62 | 2.25 | -0.14 |
|  | *Precipitation Seasonality* | -0.95 | -0.16 | 0.36 | 0.83 | 1.71 | 0.71 |
|  | *Seasonality of Vegetation Productivity* | -0.05 | 0.07 | 0.14 | 0.2 | 0.37 | 0.38 |
| **Ovine** | *Intercept* | 0.82 | 1.17 | 1.32 | 1.51 | 2.38 | 1.35 |
|  | *Annual Mean Temperature* | -0.87 | -0.57 | -0.49 | -0.44 | 0.46 | -0.43 |
|  | *Annual Precipitation* | -1.2 | -0.45 | -0.39 | -0.31 | 0.58 | -0.38 |
|  | *Precipitation Seasonality* | -0.72 | 0.1 | 0.31 | 0.52 | 1.11 | 0.35 |
|  | *Seasonality of Vegetation Productivity* | -0.15 | -0.05 | 0.05 | 0.16 | 0.33 | 0.09 |
| **Caprine** | *Intercept* | 0.24 | 0.43 | 0.57 | 0.69 | 0.96 | 0.56 |
|  | *Annual Mean Temperature* | -0.56 | -0.47 | -0.39 | -0.32 | -0.1 | -0.43 |
|  | *Annual Precipitation* | -0.34 | -0.2 | -0.09 | -0.01 | 0.24 | -0.17 |
|  | *Precipitation Seasonality* | -0.11 | 0.05 | 0.22 | 0.32 | 0.54 | 0.26 |
|  | *Seasonality of Vegetation Productivity* | -0.2 | -0.1 | -0.06 | 0.03 | 0.1 | 0.01 |
| **Equid** | *Intercept* | -1.55 | -0.32 | 0.13 | 0.25 | 0.88 | 0.7 |
|  | *Annual Mean Temperature* | -0.91 | -0.59 | -0.01 | 0.16 | 0.23 | 0.05 |
|  | *Annual Precipitation* | -2.6 | -0.96 | 0.23 | 0.42 | 0.58 | 0.13 |
|  | *Precipitation Seasonality* | -0.6 | -0.41 | -0.05 | 1.6 | 2.33 | 0.36 |
|  | *Seasonality of Vegetation Productivity* | -0.31 | -0.17 | 0.05 | 0.1 | 0.19 | 0.14 |
| **Porcine** | *Intercept* | 0.15 | 0.31 | 0.41 | 0.49 | 1.06 | 0.43 |
|  | *Annual Mean Temperature* | 0.04 | 0.12 | 0.2 | 0.31 | 0.63 | 0.17 |
|  | *Annual Precipitation* | 0.13 | 0.21 | 0.29 | 0.42 | 1.19 | 0.23 |
|  | *Precipitation Seasonality* | -0.41 | -0.06 | 0.05 | 0.17 | 0.39 | 0.16 |
|  | *Seasonality of Vegetation Productivity* | -0.05 | 0.01 | 0.09 | 0.15 | 0.21 | 0.14 |

Table S10. Summary of GWR coefficient estimates *(β)* for present distribution of livestock autochthonous breed richness (total, bovine, ovine, caprine, equid and porcine) sampled at 10 × 10 km UTM cell, using 10% of the data as bandwidth.

| **Present** | **Bandwidth = 10%** | **Min.** | **1st Q.** | **Median** | **3rd Q.** | **Max.** | **Global** |
| --- | --- | --- | --- | --- | --- | --- | --- |
| **Total Breeds** | *Intercept* | 1.91 | 3.63 | 4.52 | 5.15 | 10.58 | 4.66 |
|  | *Annual Mean Temperature* | -0.3 | 0.3 | 0.78 | 1.16 | 3.18 | 0.61 |
|  | *Annual Precipitation* | -0.47 | 0.75 | 1.09 | 1.7 | 7.76 | 0.88 |
|  | *Precipitation Seasonality* | -3.46 | -0.91 | -0.03 | 1.11 | 2.37 | 0.95 |
|  | *Seasonality of Vegetation Productivity* | 0.06 | 0.43 | 0.77 | 1.02 | 1.48 | 1.02 |
| **Bovine** | *Intercept* | 0.4 | 1.35 | 1.79 | 2.33 | 4.53 | 1.62 |
|  | *Annual Mean Temperature* | -0.28 | -0.04 | 0.06 | 0.41 | 2.07 | 0.03 |
|  | *Annual Precipitation* | 0.09 | 0.39 | 0.63 | 1.33 | 4.39 | 0.49 |
|  | *Precipitation Seasonality* | -2.32 | -0.27 | 0.05 | 0.47 | 1.07 | 0.49 |
|  | *Seasonality of Vegetation Productivity* | 0.02 | 0.19 | 0.39 | 0.57 | 0.8 | 0.49 |
| **Ovine** | *Intercept* | 0.52 | 0.67 | 0.82 | 0.96 | 2.22 | 0.78 |
|  | *Annual Mean Temperature* | -0.26 | 0 | 0.1 | 0.22 | 0.68 | 0.01 |
|  | *Annual Precipitation* | -0.36 | -0.07 | 0.05 | 0.18 | 1.73 | -0.02 |
|  | *Precipitation Seasonality* | -1.16 | -0.43 | -0.18 | 0.06 | 0.18 | -0.06 |
|  | *Seasonality of Vegetation Productivity* | -0.08 | 0 | 0.07 | 0.13 | 0.28 | 0.1 |
| **Caprine** | *Intercept* | -0.05 | 0.2 | 0.28 | 0.36 | 1.08 | 0.47 |
|  | *Annual Mean Temperature* | -0.59 | -0.24 | 0 | 0.05 | 0.19 | 0.01 |
|  | *Annual Precipitation* | -1.13 | -0.15 | 0.13 | 0.18 | 0.44 | 0.06 |
|  | *Precipitation Seasonality* | -0.14 | -0.04 | 0.1 | 0.38 | 0.84 | 0.19 |
|  | *Seasonality of Vegetation Productivity* | -0.2 | -0.07 | -0.04 | 0.05 | 0.12 | -0.01 |
| **Equid** | *Intercept* | 0.65 | 0.98 | 1.19 | 1.35 | 1.53 | 1.47 |
|  | *Annual Mean Temperature* | -0.54 | -0.02 | 0.36 | 0.58 | 0.72 | 0.45 |
|  | *Annual Precipitation* | -1.02 | -0.24 | 0.23 | 0.46 | 0.65 | 0.25 |
|  | *Precipitation Seasonality* | -0.72 | -0.5 | 0.02 | 0.59 | 1.08 | 0.08 |
|  | *Seasonality of Vegetation Productivity* | 0.04 | 0.19 | 0.22 | 0.27 | 0.37 | 0.32 |
| **Porcine** | *Intercept* | 0.03 | 0.17 | 0.26 | 0.41 | 2.02 | 0.31 |
|  | *Annual Mean Temperature* | -0.01 | 0.02 | 0.11 | 0.3 | 0.83 | 0.12 |
|  | *Annual Precipitation* | -0.21 | 0.03 | 0.07 | 0.24 | 2.15 | 0.1 |
|  | *Precipitation Seasonality* | -1.05 | 0.02 | 0.11 | 0.18 | 0.66 | 0.24 |
|  | *Seasonality of Vegetation Productivity* | 0 | 0.03 | 0.08 | 0.12 | 0.19 | 0.11 |

Table S11. Summary of GWR coefficient estimates *(β)* for past distribution of livestock autochthonous breed richness (total, bovine, ovine, caprine, equid and porcine) sampled at 10 × 10 km UTM cell, using 20% of the data as bandwidth.

| **Past** | **Bandwidth = 20%** | **Min.** | **1st Q.** | **Median** | **3rd Q.** | **Max.** | **Global** |
| --- | --- | --- | --- | --- | --- | --- | --- |
| **Total Breeds** | *Intercept* | 3.13 | 3.72 | 3.95 | 4.12 | 4.65 | 4.36 |
|  | *Annual Mean Temperature* | -1.36 | -1.01 | -0.83 | -0.77 | -0.4 | -0.98 |
|  | *Annual Precipitation* | -0.88 | -0.52 | 0.08 | 0.68 | 0.97 | -0.32 |
|  | *Precipitation Seasonality* | -0.13 | 0.92 | 1.78 | 2.43 | 3.11 | 1.84 |
|  | *Seasonality of Vegetation Productivity* | 0.04 | 0.31 | 0.43 | 0.55 | 0.77 | 0.76 |
| **Bovine** | *Intercept* | 0.79 | 1.11 | 1.2 | 1.35 | 1.56 | 1.32 |
|  | *Annual Mean Temperature* | -0.53 | -0.39 | -0.27 | -0.05 | 0.39 | -0.34 |
|  | *Annual Precipitation* | -0.35 | -0.13 | 0.24 | 0.5 | 0.73 | -0.14 |
|  | *Precipitation Seasonality* | -0.22 | 0.34 | 0.57 | 0.83 | 1.2 | 0.71 |
|  | *Seasonality of Vegetation Productivity* | 0.11 | 0.21 | 0.23 | 0.27 | 0.36 | 0.38 |
| **Ovine** | *Intercept* | 1.26 | 1.34 | 1.37 | 1.39 | 1.49 | 1.35 |
|  | *Annual Mean Temperature* | -0.53 | -0.49 | -0.47 | -0.44 | -0.17 | -0.43 |
|  | *Annual Precipitation* | -0.54 | -0.41 | -0.38 | -0.33 | -0.26 | -0.38 |
|  | *Precipitation Seasonality* | 0.15 | 0.28 | 0.36 | 0.43 | 0.57 | 0.35 |
|  | *Seasonality of Vegetation Productivity* | -0.07 | 0.01 | 0.07 | 0.14 | 0.22 | 0.09 |
| **Caprine** | *Intercept* | 0.42 | 0.54 | 0.58 | 0.62 | 0.7 | 0.56 |
|  | *Annual Mean Temperature* | -0.51 | -0.47 | -0.41 | -0.38 | -0.33 | -0.43 |
|  | *Annual Precipitation* | -0.24 | -0.18 | -0.12 | -0.08 | -0.02 | -0.17 |
|  | *Precipitation Seasonality* | 0.13 | 0.21 | 0.26 | 0.3 | 0.33 | 0.26 |
|  | *Seasonality of Vegetation Productivity* | -0.1 | -0.05 | -0.02 | 0.03 | 0.06 | 0.01 |
| **Equid** | *Intercept* | -0.46 | 0.28 | 0.36 | 0.49 | 0.82 | 0.7 |
|  | *Annual Mean Temperature* | -0.62 | -0.16 | 0.09 | 0.17 | 0.21 | 0.05 |
|  | *Annual Precipitation* | -1.32 | -0.08 | 0.24 | 0.43 | 0.53 | 0.13 |
|  | *Precipitation Seasonality* | -0.35 | -0.17 | 0.14 | 0.89 | 1.69 | 0.36 |
|  | *Seasonality of Vegetation Productivity* | -0.25 | -0.14 | 0.08 | 0.13 | 0.2 | 0.14 |
| **Porcine** | *Intercept* | 0.32 | 0.39 | 0.44 | 0.45 | 0.6 | 0.43 |
|  | *Annual Mean Temperature* | 0.09 | 0.15 | 0.18 | 0.24 | 0.34 | 0.17 |
|  | *Annual Precipitation* | 0.2 | 0.24 | 0.27 | 0.31 | 0.6 | 0.23 |
|  | *Precipitation Seasonality* | 0 | 0.08 | 0.13 | 0.18 | 0.25 | 0.16 |
|  | *Seasonality of Vegetation Productivity* | 0.03 | 0.07 | 0.12 | 0.15 | 0.19 | 0.14 |

Table S12. Summary of GWR coefficient estimates *(β)* for present distribution of livestock autochthonous breed richness (total, bovine, ovine, caprine, equid and porcine) sampled at 10 × 10 km UTM cell, using 20% of the data as bandwidth.

| **Present** | **Bandwidth = 20%** | **Min.** | **1st Q.** | **Median** | **3rd Q.** | **Max.** | **Global** |
| --- | --- | --- | --- | --- | --- | --- | --- |
| **Total Breeds** | *Intercept* | 3.5 | 4.38 | 4.72 | 4.89 | 6.09 | 4.66 |
|  | *Annual Mean Temperature* | 0.19 | 0.43 | 0.65 | 0.9 | 1.2 | 0.61 |
|  | *Annual Precipitation* | 0.48 | 0.85 | 1.09 | 1.27 | 2.27 | 0.88 |
|  | *Precipitation Seasonality* | -0.19 | 0.39 | 0.75 | 1.12 | 1.48 | 0.95 |
|  | *Seasonality of Vegetation Productivity* | 0.39 | 0.68 | 0.96 | 1.13 | 1.28 | 1.02 |
| **Bovine** | *Intercept* | 1.16 | 1.57 | 1.81 | 1.92 | 2.71 | 1.62 |
|  | *Annual Mean Temperature* | -0.08 | 0 | 0.07 | 0.13 | 0.81 | 0.03 |
|  | *Annual Precipitation* | 0.37 | 0.47 | 0.57 | 0.68 | 1.92 | 0.49 |
|  | *Precipitation Seasonality* | -0.62 | 0.27 | 0.43 | 0.54 | 0.69 | 0.49 |
|  | *Seasonality of Vegetation Productivity* | 0.19 | 0.34 | 0.53 | 0.61 | 0.67 | 0.49 |
| **Ovine** | *Intercept* | 0.68 | 0.79 | 0.82 | 0.84 | 1.15 | 0.78 |
|  | *Annual Mean Temperature* | -0.1 | 0.02 | 0.1 | 0.14 | 0.3 | 0.01 |
|  | *Annual Precipitation* | -0.09 | -0.01 | 0.04 | 0.09 | 0.5 | -0.02 |
|  | *Precipitation Seasonality* | -0.4 | -0.18 | -0.09 | -0.02 | 0.07 | -0.06 |
|  | *Seasonality of Vegetation Productivity* | -0.01 | 0.04 | 0.08 | 0.14 | 0.18 | 0.1 |
| **Caprine** | *Intercept* | 0.29 | 0.33 | 0.38 | 0.43 | 0.54 | 0.47 |
|  | *Annual Mean Temperature* | -0.34 | -0.09 | 0.01 | 0.06 | 0.15 | 0.01 |
|  | *Annual Precipitation* | -0.41 | -0.07 | 0.09 | 0.16 | 0.24 | 0.06 |
|  | *Precipitation Seasonality* | 0 | 0.06 | 0.16 | 0.33 | 0.52 | 0.19 |
|  | *Seasonality of Vegetation Productivity* | -0.13 | -0.06 | -0.04 | 0.01 | 0.07 | -0.01 |
| **Equid** | *Intercept* | 1.09 | 1.27 | 1.36 | 1.41 | 1.5 | 1.47 |
|  | *Annual Mean Temperature* | -0.13 | 0.17 | 0.42 | 0.56 | 0.65 | 0.45 |
|  | *Annual Precipitation* | -0.5 | 0 | 0.25 | 0.43 | 0.56 | 0.25 |
|  | *Precipitation Seasonality* | -0.41 | -0.22 | 0.04 | 0.41 | 0.78 | 0.08 |
|  | *Seasonality of Vegetation Productivity* | 0.19 | 0.22 | 0.25 | 0.31 | 0.37 | 0.32 |
| **Porcine** | *Intercept* | 0.15 | 0.25 | 0.32 | 0.36 | 0.72 | 0.31 |
|  | *Annual Mean Temperature* | 0.01 | 0.05 | 0.13 | 0.2 | 0.36 | 0.12 |
|  | *Annual Precipitation* | 0.04 | 0.06 | 0.08 | 0.11 | 0.65 | 0.1 |
|  | *Precipitation Seasonality* | -0.12 | 0.17 | 0.21 | 0.24 | 0.34 | 0.24 |
|  | *Seasonality of Vegetation Productivity* | 0.02 | 0.06 | 0.11 | 0.15 | 0.18 | 0.11 |


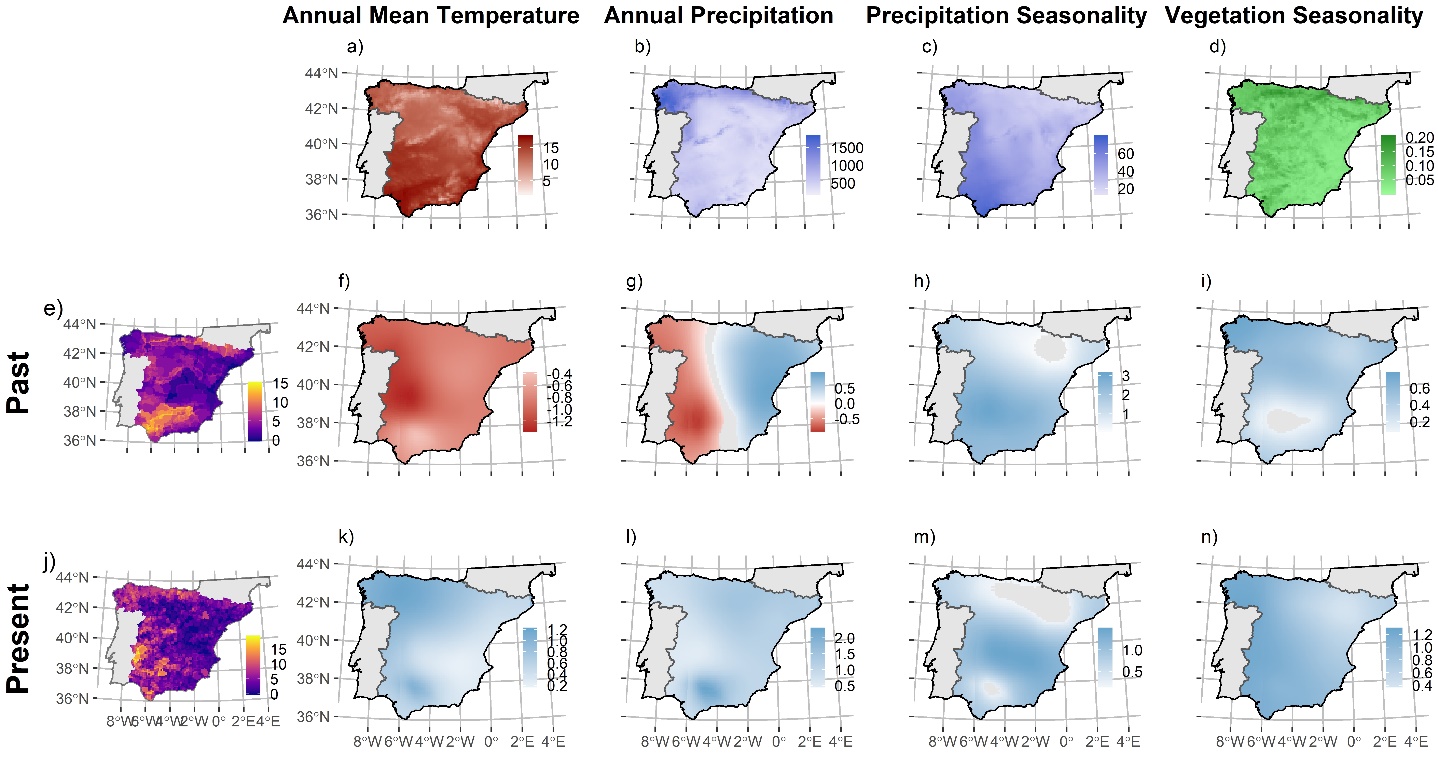


Figure S1: Maps of regression coefficients (surface of predictions) resulting from Geographically Weighted Regression models using 20% of the data as bandwidth, fitting the relationships between total autochthonous breed richness -sampled at 10×10 km UTM grid cell- for past (e) and present (n) distributions, using as predictors annual mean temperature (a), annual precipitation (b), precipitation seasonality (c) and vegetation productivity seasonality (d). Depicted coefficients are only coloured when statistically significant at [*P* = 0] ≤ 0.05. Blue colour represents positive coefficients and red colour represents negative associations. Figure was created using “sf” and “ggplot2” packages in R v3.6.0 software (<https://www.R-project.org/>).

**
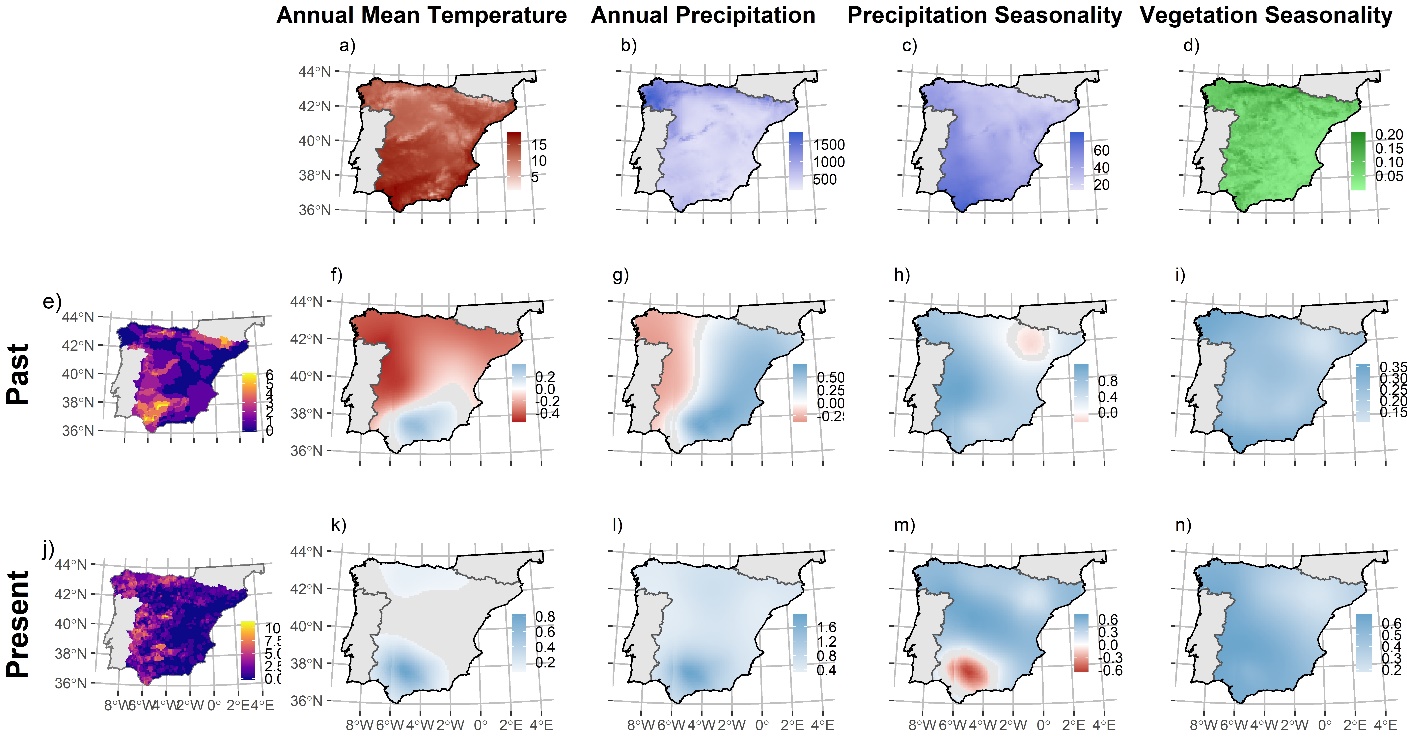
**

Figure S2: Maps of regression coefficients (surface of predictions) resulting from Geographically Weighted Regression models using 20% of the data as bandwidth, fitting the relationships between bovine autochthonous breed richness -sampled at 10×10 km UTM grid cell- for past (e) and present (n) distributions, using as predictors annual mean temperature (a), annual precipitation (b), precipitation seasonality (c) and vegetation productivity seasonality (d). Depicted coefficients are only coloured when statistically significant at [*P* = 0] ≤ 0.05. Blue colour represents positive coefficients and red colour represents negative associations. Figure was created using “sf” and “ggplot2” packages in R v3.6.0 software (<https://www.R-project.org/>).


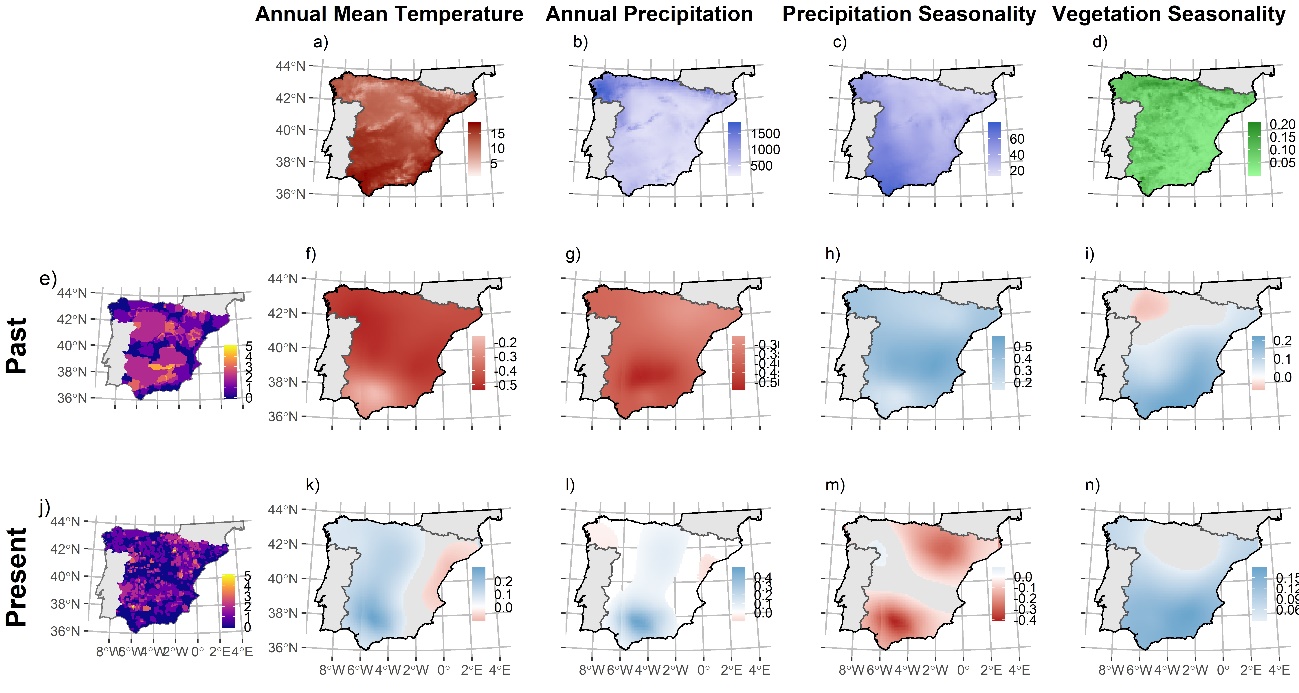
Figure S3: Maps of regression coefficients (surface of predictions) resulting from Geographically Weighted Regression models using 20% of the data as bandwidth, fitting the relationships between ovine autochthonous breed richness -sampled at 10×10 km UTM grid cell- for past (e) and present (n) distributions, using as predictors annual mean temperature (a), annual precipitation (b), precipitation seasonality (c) and vegetation productivity seasonality (d). Depicted coefficients are only coloured when statistically significant at [*P* = 0] ≤ 0.05. Blue colour represents positive coefficients and red colour represents negative associations. Figure was created using “sf” and “ggplot2” packages in R v3.6.0 software (<https://www.R-project.org/>).


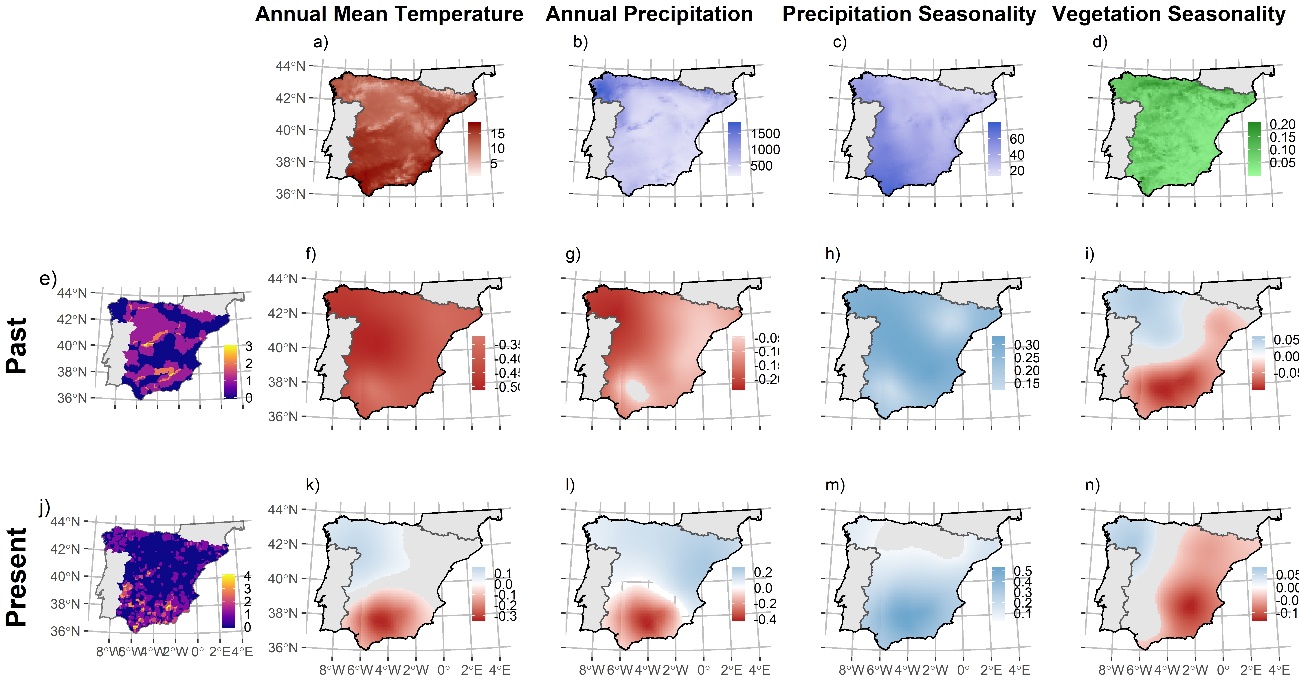


Figure S4: Maps of regression coefficients (surface of predictions) resulting from Geographically Weighted Regression models using 20% of the data as bandwidth, fitting the relationships between caprine autochthonous breed richness -sampled at 10×10 km UTM grid cell- for past (e) and present (n) distributions, using as predictors annual mean temperature (a), annual precipitation (b), precipitation seasonality (c) and vegetation productivity seasonality (d). Depicted coefficients are only coloured when statistically significant at [*P* = 0] ≤ 0.05. Blue colour represents positive coefficients and red colour represents negative associations. Figure was created using “sf” and “ggplot2” packages in R v3.6.0 software (<https://www.R-project.org/>).


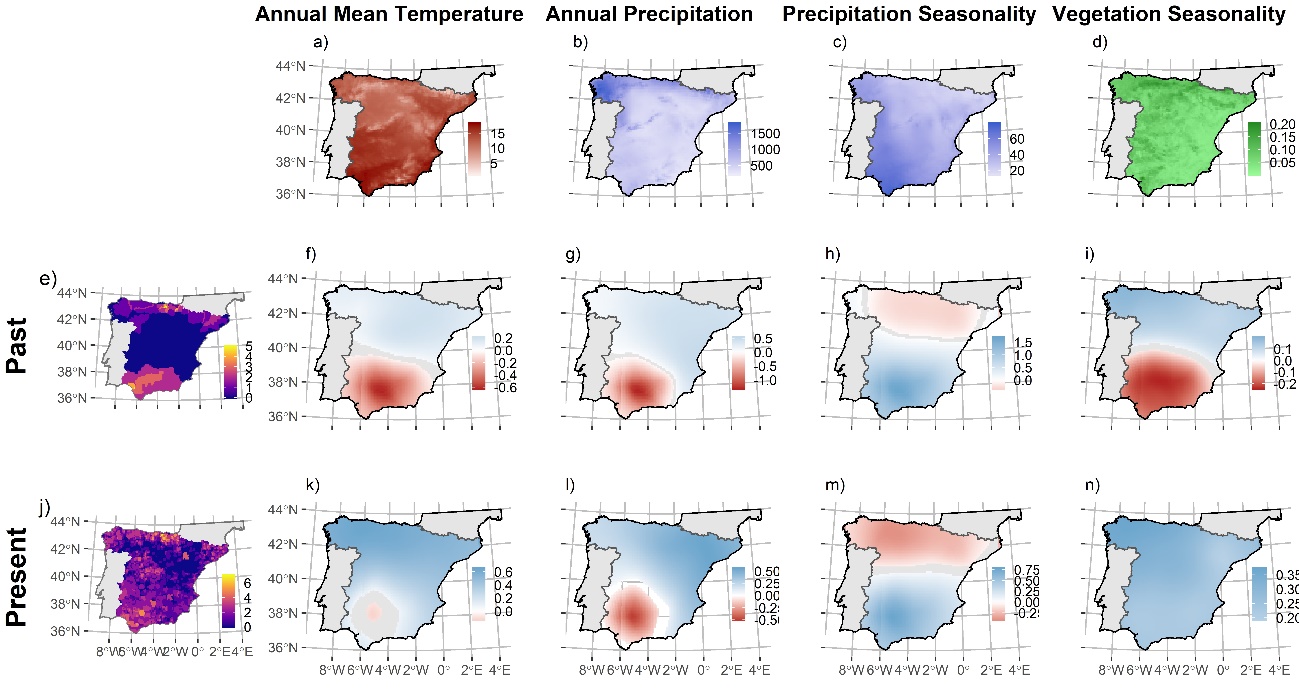
Figure S5: Maps of regression coefficients (surface of predictions) resulting from Geographically Weighted Regression models using 20% of the data as bandwidth, fitting the relationships between equine autochthonous breed richness -sampled at 10×10 km UTM grid cell- for past (e) and present (n) distributions, using as predictors annual mean temperature (a), annual precipitation (b), precipitation seasonality (c) and vegetation productivity seasonality (d). Depicted coefficients are only coloured when statistically significant at [*P* = 0] ≤ 0.05. Blue colour represents positive coefficients and red colour represents negative associations. Figure was created using “sf” and “ggplot2” packages in R v3.6.0 software (<https://www.R-project.org/>).


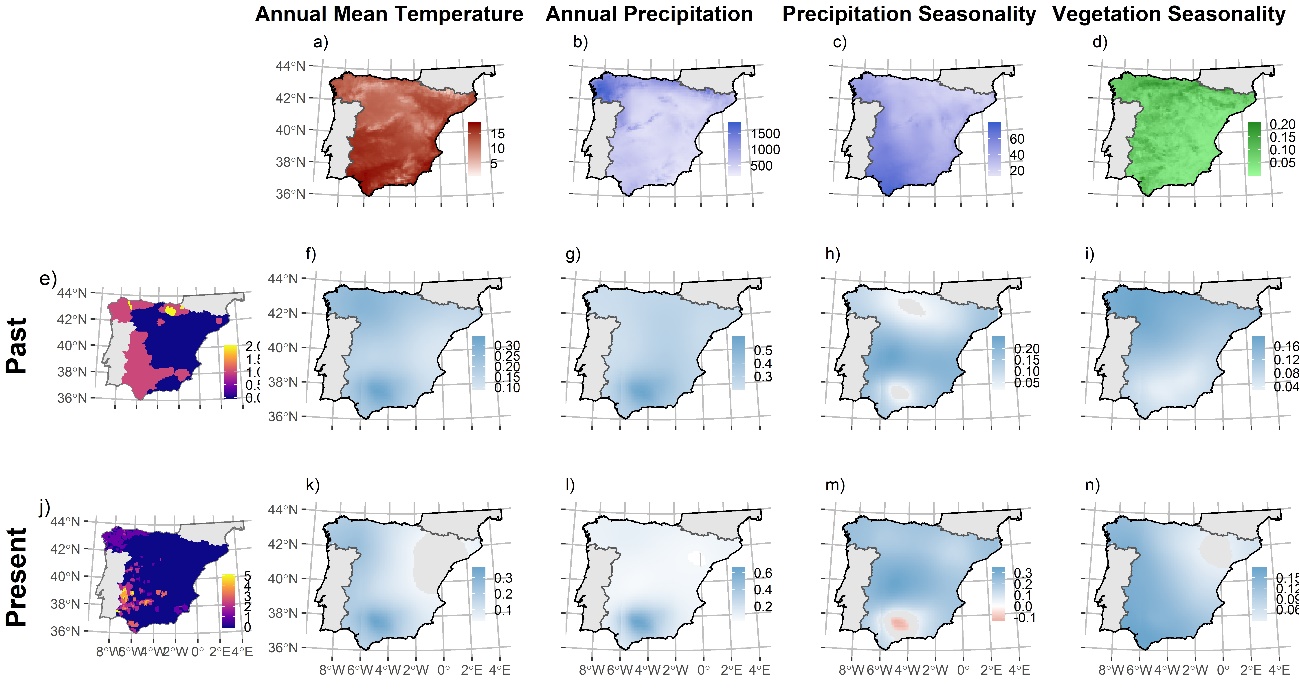
Figure S6: Maps of regression coefficients (surface of predictions) resulting from Geographically Weighted Regression models using 20% of the data as bandwidth, fitting the relationships between porcine autochthonous breed richness -sampled at 10×10 km UTM grid cell- for past (e) and present (n) distributions, using as predictors annual mean temperature (a), annual precipitation (b), precipitation seasonality (c) and vegetation productivity seasonality (d). Depicted coefficients are only coloured when statistically significant at [*P* = 0] ≤ 0.05. Blue colour represents positive coefficients and red colour represents negative associations. Figure was created using “sf” and “ggplot2” packages in R v3.6.0 software (<https://www.R-project.org/>).

1. **Sensitivity Analysis**

Table S13: Quasi-global R^2^ from the Geographically Weighted Regression (analyses extent: 5% of data) fitted models performed at 10% of the total data analysis extent of the local breed richness, i.e. autochthonous, removing extinct and new recognised breeds (bovine, ovine, caprine, equid -hoses and donkeys-, porcine species and total, sampled at 10×10 km UTM grid cell) for past and present distributions, using as predictors annual mean temperature, annual precipitation, precipitation seasonality and vegetation productivity seasonality.

|  | **Global Quasi-R2** | |
| --- | --- | --- |
|  | ***Past*** | ***Present*** |
| **Bovine** | 0.59 | 0.45 |
| **Ovine** | 0.40 | 0.20 |
| **Caprine** | 0.40 | 0.29 |
| **Equid** | 0.73 | 0.41 |
| **Porcine** | 0.72 | 0.37 |
| **Total** | 0.62 | 0.45 |

Table S14. Summary of GWR coefficient estimates *(β)* for past distribution of livestock local breed richness, i.e. autochthonous, removing extinct and new recognised breeds (total, bovine, ovine, caprine, equid and porcine) sampled at 10 × 10 km UTM cell, using 5% of the data as bandwidth.

| **Past** | **Sensitivity Analysis** | **Min.** | **1st Q.** | **Median** | **3rd Q.** | **Max.** | **Global** |
| --- | --- | --- | --- | --- | --- | --- | --- |
| **Total Breeds** | *Intercept* | 0.48 | 1.89 | 2.85 | 3.93 | 11.65 | 4.20 |
|  | *Annual Mean Temperature* | -2.84 | -1.43 | -0.94 | -0.63 | 3.68 | -0.90 |
|  | *Annual Precipitation* | -3.39 | -0.78 | -0.01 | 0.65 | 8.05 | -0.36 |
|  | *Precipitation Seasonality* | -3.90 | -1.11 | 0.68 | 2.32 | 5.94 | 1.71 |
|  | *Seasonality of Vegetation Productivity* | -0.85 | -0.13 | 0.09 | 0.34 | 0.81 | 0.67 |
| **Bovine** | *Intercept* | -0.49 | 0.34 | 0.92 | 1.36 | 4.39 | 1.20 |
|  | *Annual Mean Temperature* | -1.32 | -0.44 | -0.24 | 0.02 | 2.55 | -0.27 |
|  | *Annual Precipitation* | -1.75 | -0.24 | 0.20 | 0.63 | 3.91 | -0.16 |
|  | *Precipitation Seasonality* | -2.25 | -0.70 | 0.08 | 0.75 | 1.82 | 0.57 |
|  | *Seasonality of Vegetation Productivity* | -0.22 | -0.06 | 0.05 | 0.16 | 0.52 | 0.31 |
| **Ovine** | *Intercept* | 0.04 | 1.02 | 1.32 | 1.81 | 4.30 | 1.34 |
|  | *Annual Mean Temperature* | -1.47 | -0.60 | -0.45 | -0.32 | 1.28 | -0.41 |
|  | *Annual Precipitation* | -2.60 | -0.54 | -0.40 | -0.16 | 2.92 | -0.39 |
|  | *Precipitation Seasonality* | -2.09 | -0.05 | 0.20 | 0.58 | 2.13 | 0.34 |
|  | *Seasonality of Vegetation Productivity* | -0.18 | -0.09 | 0.04 | 0.18 | 0.45 | 0.09 |
| **Caprine** | *Intercept* | -0.17 | 0.30 | 0.60 | 0.77 | 1.47 | 0.56 |
|  | *Annual Mean Temperature* | -0.80 | -0.45 | -0.36 | -0.27 | 0.44 | -0.42 |
|  | *Annual Precipitation* | -1.08 | -0.25 | -0.04 | 0.09 | 0.91 | -0.17 |
|  | *Precipitation Seasonality* | -0.44 | -0.11 | 0.10 | 0.35 | 1.03 | 0.25 |
|  | *Seasonality of Vegetation Productivity* | -0.33 | -0.13 | -0.09 | 0.03 | 0.18 | 0.01 |
| **Equid** | *Intercept* | -2.49 | -0.37 | 0.08 | 0.18 | 0.79 | 0.70 |
|  | *Annual Mean Temperature* | -1.12 | -0.42 | -0.06 | 0.11 | 0.25 | 0.05 |
|  | *Annual Precipitation* | -3.26 | -1.30 | 0.13 | 0.35 | 0.63 | 0.13 |
|  | *Precipitation Seasonality* | -0.87 | -0.48 | -0.17 | 1.48 | 2.73 | 0.36 |
|  | *Seasonality of Vegetation Productivity* | -0.37 | -0.15 | 0.00 | 0.09 | 0.20 | 0.14 |
| **Porcine** | *Intercept* | -0.18 | 0.21 | 0.33 | 0.55 | 1.65 | 0.40 |
|  | *Annual Mean Temperature* | -0.16 | 0.06 | 0.17 | 0.33 | 0.87 | 0.15 |
|  | *Annual Precipitation* | -0.55 | 0.15 | 0.29 | 0.45 | 1.93 | 0.23 |
|  | *Precipitation Seasonality* | -0.85 | -0.11 | 0.03 | 0.17 | 0.64 | 0.19 |
|  | *Seasonality of Vegetation Productivity* | -0.12 | -0.01 | 0.03 | 0.11 | 0.24 | 0.11 |

Table S15. Summary of GWR coefficient estimates *(β)* for present distribution of local livestock breed richness i.e. autochthonous, removing extinct and new recognised breeds (total, bovine, ovine, caprine, equid and porcine) sampled at 10 × 10 km UTM cell, using 5% of the data as bandwidth.

| **Present** | **Sensitivity Analysis** | **Min.** | **1st Q.** | **Median** | **3rd Q.** | **Max.** | **Global** |
| --- | --- | --- | --- | --- | --- | --- | --- |
| **Total Breeds** | *Intercept* | 1.21 | 2.54 | 3.88 | 5.35 | 11.21 | 4.52 |
|  | *Annual Mean Temperature* | -1.31 | 0.03 | 0.64 | 1.16 | 4.09 | 0.59 |
|  | *Annual Precipitation* | -1.45 | 0.41 | 1.07 | 2.17 | 8.13 | 0.91 |
|  | *Precipitation Seasonality* | -3.97 | -1.67 | -0.96 | 1.02 | 3.22 | 0.77 |
|  | *Seasonality of Vegetation Productivity* | -0.08 | 0.18 | 0.53 | 0.98 | 1.65 | 0.94 |
| **Bovine** | *Intercept* | 0.01 | 0.94 | 1.52 | 2.69 | 5.31 | 1.62 |
|  | *Annual Mean Temperature* | -0.78 | -0.17 | 0.06 | 0.63 | 2.79 | 0.02 |
|  | *Annual Precipitation* | -0.51 | 0.35 | 0.69 | 1.93 | 5.25 | 0.48 |
|  | *Precipitation Seasonality* | -2.83 | -0.64 | -0.28 | 0.43 | 1.93 | 0.5 |
|  | *Seasonality of Vegetation Productivity* | -0.12 | 0.04 | 0.24 | 0.56 | 1 | 0.5 |
| **Ovine** | *Intercept* | 0.21 | 0.57 | 0.8 | 1.1 | 2.9 | 0.78 |
|  | *Annual Mean Temperature* | -0.46 | -0.06 | 0.09 | 0.27 | 0.99 | 0.01 |
|  | *Annual Precipitation* | -0.71 | -0.2 | 0.02 | 0.27 | 2.55 | -0.02 |
|  | *Precipitation Seasonality* | -1.55 | -0.59 | -0.19 | 0.14 | 0.54 | -0.06 |
|  | *Seasonality of Vegetation Productivity* | -0.18 | -0.01 | 0.05 | 0.14 | 0.36 | 0.1 |
| **Caprine** | *Intercept* | -0.38 | 0.12 | 0.21 | 0.32 | 1.67 | 0.47 |
|  | *Annual Mean Temperature* | -0.87 | -0.26 | 0 | 0.06 | 0.44 | 0.01 |
|  | *Annual Precipitation* | -1.75 | -0.21 | 0.16 | 0.24 | 1.24 | 0.06 |
|  | *Precipitation Seasonality* | -0.51 | -0.12 | -0.01 | 0.42 | 1.03 | 0.19 |
|  | *Seasonality of Vegetation Productivity* | -0.26 | -0.07 | -0.02 | 0.05 | 0.17 | -0.01 |
| **Equid** | *Intercept* | 0.03 | 0.64 | 1.01 | 1.29 | 2.11 | 1.47 |
|  | *Annual Mean Temperature* | -0.77 | 0.07 | 0.34 | 0.58 | 0.95 | 0.45 |
|  | *Annual Precipitation* | -1.75 | -0.47 | 0.21 | 0.51 | 0.87 | 0.25 |
|  | *Precipitation Seasonality* | -1.4 | -0.69 | 0.16 | 0.62 | 1.42 | 0.08 |
|  | *Seasonality of Vegetation Productivity* | 0.02 | 0.13 | 0.21 | 0.27 | 0.34 | 0.32 |
| **Porcine** | *Intercept* | -0.15 | 0.06 | 0.14 | 0.23 | 1.27 | 0.18 |
|  | *Annual Mean Temperature* | -0.23 | 0.01 | 0.04 | 0.21 | 0.4 | 0.1 |
|  | *Annual Precipitation* | -0.41 | 0.01 | 0.04 | 0.26 | 1.2 | 0.14 |
|  | *Precipitation Seasonality* | -0.61 | -0.19 | 0.03 | 0.11 | 0.31 | 0.07 |
|  | *Seasonality of Vegetation Productivity* | -0.02 | 0 | 0.02 | 0.06 | 0.12 | 0.03 |

**Appendix 3. Ordination Logistic Regression Models**


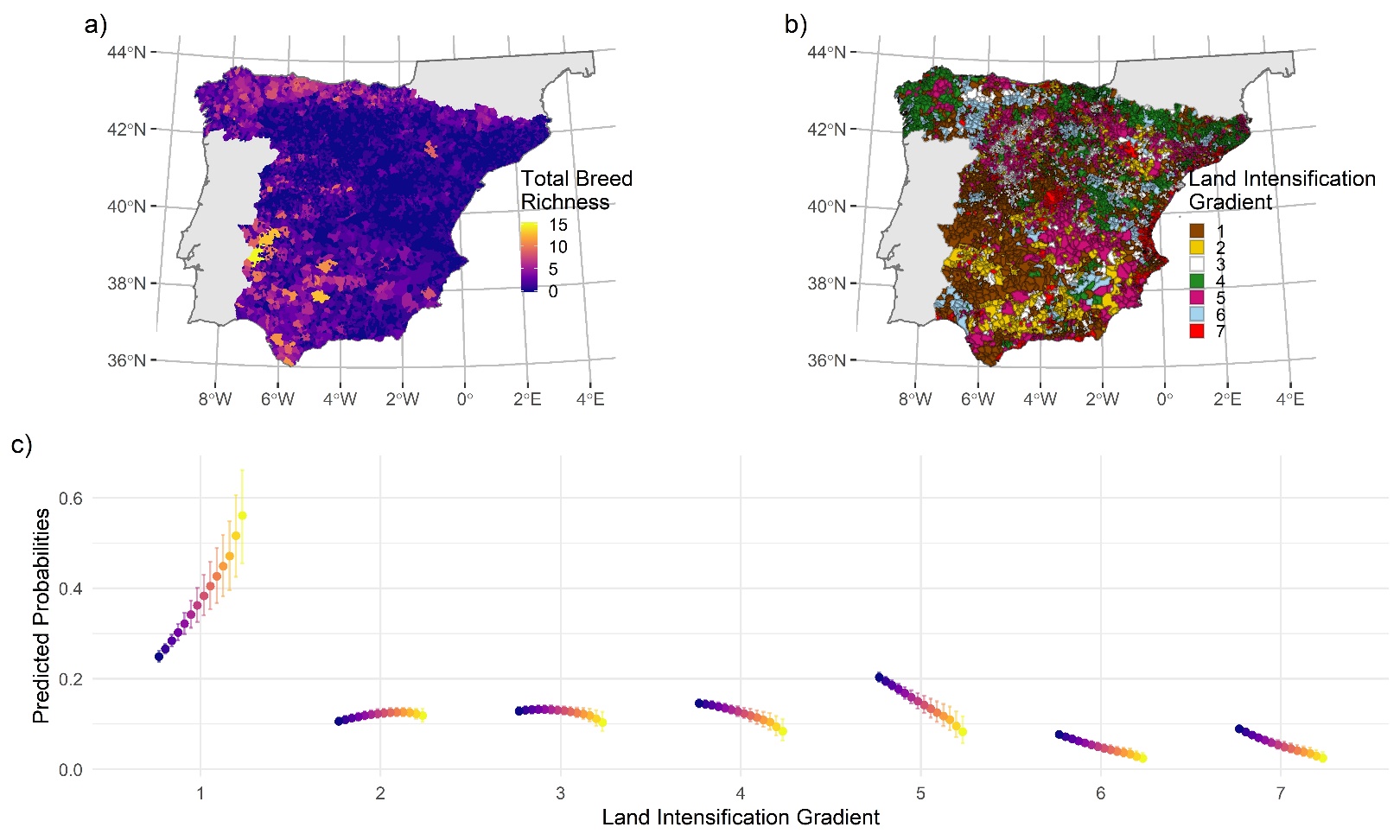


Figure S1. Maps by municipalities of total autochthonous breed richness distribution (a), and land cover transition (intensification gradient from 1 to 7) (b). Figure was created using “sf” and “ggplot2” packages in R v3.6.0 software (<https://www.R-project.org/>).

Gradient Definition:

1. Abandonment: those municipalities where afforestation dominates, related to agricultural abandonment in tension with conversion to agriculture.

2.Extensification: agriculture extensification.

3.Stability: municipalities where stability along the 22 years period dominates.

4.Forest Changes: afforestation and changes on forest composition.

5. Intensification: agricultural intensification.

6. Deforestation: deforestation.

7.Artificialization: increase of urban areas.

Tables S1. Summary of OLR relating contemporary total autochthonous livestock breed richness distribution and land cover transition.

|  | Estimate | Std. Error | z value | Pr(>\|z\|) |
| --- | --- | --- | --- | --- |
| Total Breed Richness | -0.08993 | 0.01337 | -6.727 | 1.74E-11 |

| Threshold coefficients: | | | |
| --- | --- | --- | --- |
|  | Estimate | Std. Error | z value |
| 1\|2 | -1.10362 | 0.02819 | -39.144 |
| 2\|3 | -0.59673 | 0.02597 | -22.981 |
| 3\|4 | -0.06384 | 0.02519 | -2.534 |
| 4\|5 | 0.53147 | 0.02603 | 20.416 |
| 5\|6 | 1.60895 | 0.03257 | 49.407 |
| 6\|7 | 2.32004 | 0.04168 | 55.667 |

Tables S2. Summary of OLR relating contemporary bovine autochthonous livestock breed richness distribution and land cover transition.

|  | Estimate | Std. Error | z value | Pr(>\|z\|) |
| --- | --- | --- | --- | --- |
| Bovine Richness | -0.2475 | 0.02652 | -9.332 | <2e-16 |

| Threshold coefficients: | | | |
| --- | --- | --- | --- |
|  | Estimate | Std. Error | z value |
| 1\|2 | -1.10097 | 0.0268 | -41.08 |
| 2\|3 | -0.59164 | 0.02446 | -24.19 |
| 3\|4 | -0.0574 | 0.02362 | -2.43 |
| 4\|5 | 0.53967 | 0.02449 | 22.04 |
| 5\|6 | 1.61845 | 0.03142 | 51.51 |
| 6\|7 | 2.32971 | 0.04081 | 57.09 |

Tables S3. Summary of OLR relating contemporary ovine autochthonous livestock breed richness distribution and land cover transition.

|  | Estimate | Std. Error | z value | Pr(>\|z\|) |
| --- | --- | --- | --- | --- |
| Ovine Richness | -0.14331 | 0.04695 | -3.053 | 0.00227 |

| Threshold coefficients: | | | |
| --- | --- | --- | --- |
|  | Estimate | Std. Error | z value |
| 1\|2 | -1.044425 | 0.026445 | -39.494 |
| 2\|3 | -0.54037 | 0.024307 | -22.231 |
| 3\|4 | -0.008385 | 0.023587 | -0.356 |
| 4\|5 | 0.586376 | 0.024552 | 23.883 |
| 5\|6 | 1.661827 | 0.031508 | 52.743 |
| 6\|7 | 2.372679 | 0.040858 | 58.071 |

Tables S4. Summary of OLR relating contemporary caprine autochthonous livestock breed richness distribution and land cover transition.

|  | Estimate | Std. Error | z value | Pr(>\|z\|) |
| --- | --- | --- | --- | --- |
| Caprine Richness | -0.28914 | 0.07134 | -4.053 | 5.06E-05 |

| Threshold coefficients: | | | |
| --- | --- | --- | --- |
|  | Estimate | Std. Error | z value |
| 1\|2 | -1.042902 | 0.025873 | -40.309 |
| 2\|3 | -0.538139 | 0.023649 | -22.756 |
| 3\|4 | -0.005831 | 0.022889 | -0.255 |
| 4\|5 | 0.58911 | 0.02388 | 24.67 |
| 5\|6 | 1.664839 | 0.031025 | 53.661 |
| 6\|7 | 2.375543 | 0.040515 | 58.634 |

Tables S5. Summary of OLR relating contemporary equine autochthonous livestock breed richness distribution and land cover transition.

|  | Estimate | Std. Error | z value | Pr(>\|z\|) |
| --- | --- | --- | --- | --- |
| Equine Richness | 0.02468 | 0.03098 | 0.797 | 0.426 |

| Threshold coefficients: | | | |
| --- | --- | --- | --- |
|  | Estimate | Std. Error | z value |
| 1\|2 | -1.01288 | 0.02712 | -37.345 |
| 2\|3 | -0.50908 | 0.02502 | -20.347 |
| 3\|4 | 0.02296 | 0.02435 | 0.943 |
| 4\|5 | 0.61777 | 0.02538 | 24.345 |
| 5\|6 | 1.69208 | 0.03224 | 52.478 |
| 6\|7 | 2.4022 | 0.0415 | 57.887 |

Tables S6. Summary of OLR relating contemporary autochthonous porcine livestock breed richness distribution and land cover transition.

|  | Estimate | Std. Error | z value | Pr(>\|z\|) |
| --- | --- | --- | --- | --- |
| Porcine Richness | -0.31965 | 0.08291 | -3.856 | 0.000115 |

| Threshold coefficients: | | | |
| --- | --- | --- | --- |
|  | Estimate | Std. Error | z value |
| 1\|2 | -1.032406 | 0.025459 | -40.552 |
| 2\|3 | -0.527487 | 0.023215 | -22.722 |
| 3\|4 | 0.004858 | 0.022454 | 0.216 |
| 4\|5 | 0.599585 | 0.023483 | 25.533 |
| 5\|6 | 1.674476 | 0.030769 | 54.422 |
| 6\|7 | 2.384974 | 0.040331 | 59.134 |

Tables S7. Summary of OLR relating contemporary autochthonous increasing in number (see Appendix 1, Table S1) livestock breed richness distribution and land cover transition.

|  | Estimate | Std. Error | z value | Pr(>\|z\|) |
| --- | --- | --- | --- | --- |
| Increasing Number Breed Richness | -0.36364 | 0.03648 | -9.969 | <2e-16 |

| Threshold coefficients: | | | |
| --- | --- | --- | --- |
|  | Estimate | Std. Error | z value |
| 1\|2 | -1.10231 | 0.02669 | -41.3 |
| 2\|3 | -0.59159 | 0.02431 | -24.34 |
| 3\|4 | -0.056 | 0.02343 | -2.39 |
| 4\|5 | 0.54147 | 0.02431 | 22.28 |
| 5\|6 | 1.61954 | 0.03131 | 51.73 |
| 6\|7 | 2.33057 | 0.04073 | 57.22 |

Tables S8. Summary of OLR relating contemporary autochthonous in danger of extinction (see Appendix 1, Table S1) livestock breed richness distribution and land cover transition.

|  | Estimate | Std. Error | z value | Pr(>\|z\|) |
| --- | --- | --- | --- | --- |
| Increasing Number Breed Richness | -0.09707 | 0.02063 | -4.704 | 2.55E-06 |

| Threshold coefficients: | | | |
| --- | --- | --- | --- |
|  | Estimate | Std. Error | z value |
| 1\|2 | -1.06628 | 0.02709 | -39.363 |
| 2\|3 | -0.56132 | 0.02492 | -22.525 |
| 3\|4 | -0.02999 | 0.02426 | -1.236 |
| 4\|5 | 0.56472 | 0.02519 | 22.418 |
| 5\|6 | 1.64152 | 0.03193 | 51.405 |
| 6\|7 | 2.35232 | 0.0412 | 57.102 |
